# Real-world effectiveness and safety of prolonged bedaquiline course in the treatment of drug-resistant tuberculosis—a multi-center retrospective cohort study in a country with a high burden of drug-resistant tuberculosis

**DOI:** 10.1128/spectrum.00097-25

**Published:** 2025-07-07

**Authors:** Yuanyuan Yu, Jie Cao, Hongqiu Pan, Hui Zhong, Xiaocui Wu, Jinghui Yang, Liping Cheng, Qingrong Qu, Lei Wang, Fuhui Lu, Hongyu Chen, Jie Wang, Wei Sha, Qin Sun

**Affiliations:** 1 Shanghai Clinical Research Center for Infectious Disease (Tuberculosis), Shanghai Key Laboratory of Tuberculosis, Shanghai Pulmonary Hospital, Tongji University School of Medicine 481875, Shanghai, China; 2 Department of Tuberculosis, The Third People’s Hospital of Zhenjiang affiliated to Jiangsu University, Zhenjiang, China; 3 Department of Infectious Diseases, Shanghai Fengxian Guhua Hospital, Shanghai, China; 4 Department of Clinical Laboratory, Shanghai Pulmonary Hospital affiliated to Tongji University, Shanghai, China; The University of Texas at Tyler, Tyler, Texas, USA

**Keywords:** prolonged, bedaquiline, effectiveness, safety, propensity score match (PSM)

## Abstract

Bedaquiline (BDQ) has gradually become a core drug in drug-resistant tuberculosis (DR-TB) treatment, and the additional benefits of prolonging BDQ use remain unclear. Patients with DR-TB who received a BDQ-containing regimen from three Chinese clinical centers between 1 March 2018 and 31 December 2021 were retrospectively analyzed. Treatment outcomes and adverse drug reactions were compared between 6-month and prolonged BDQ treatment group before and after adjustment by propensity score matching (PSM). A total of 160 patients were enrolled, 72 patients were treated with BDQ for 6 months, and 88 patients were over 6 months, of which the median duration was 9 months (IQR: 8–11 months). After PSM adjustment, there were no significant differences in treatment outcome between the prolonged groups (7–9, 10−12, >12 months) and the 6-month group (all *P* > 0.05). A total of 35 patients met the criteria for BDQ prolongation but did not receive it, resulting in a success rate of 60%, significantly lower than the prolonged group (78.4%, *P* = 0.038); however, after adjustment by PSM, there was no statistically significance (*P* > 0.05). The median treatment duration (23 months, IQR: 18.50–25.00 months) was significantly longer than the prolonged group (18 months, IQR: 15.00–20.25 months, *P* < 0.001). Additionally, two deaths occurred in the prolonged group, and none in the 6-month group. The cause of death in one patient was adjudicated as anti-TB treatment-related, while the other one was considered not. There were no significant differences in the effectiveness and safety between 6-month and prolonged group, it’s still recommended to prolong BDQ use under close monitoring when anti-TB drugs are insufficient to form an effective treatment regimen. Prolonged use of BDQ achieved similar treatment outcomes while potentially shortening the overall anti-TB duration.

**IMPORTANCE:**

This real-world retrospective cohort study provides critical evidence on the extended application of Bedaquiline (BDQ) in managing drug-resistant tuberculosis (DR-TB). To date, the effectiveness and safety data regarding prolonged BDQ treatment are still lacking, and the additional benefits of prolonged BDQ use remain unclear. Our findings notably demonstrate that prolonged use of BDQ can achieve similar treatment success rates while potentially shortening the overall anti-TB treatment duration. We conclude that when the anti-TB drugs are insufficient to form an effective treatment regimen, prolonged BDQ use with rigorous safety monitoring is recommended. Our study significantly advances the evidence base for prolonged use of BDQ in clinical practice.

## INTRODUCTION

Tuberculosis (TB) remains a significant public health issue affecting global health. The World Health Organization (WHO) reported a treatment success rate of 63.82% for drug-resistant TB (DR-TB) in 2020; however, the success rate in China was only 50.71% (1). Bedaquiline (BDQ) is an oral diarylquinoline with potent bactericidal and sterilizing activity, functioning by inhibiting mycobacterial ATP synthase (2, 3). The BDQ clinical access program (BCAP) was conducted in South Africa from March 2013 to March 2015 (4). In patients with rifampin-resistant TB (RR-TB) and multiple drug-resistant TB (MDR-TB), the addition of BDQ for 6 months to a total treatment regimen of at least 18 months achieved a 73% success rate and significantly improved treatment outcomes. Increasing clinical data have confirmed that BDQ has gradually become a core drug in treating DR-TB (5–8). In 2018, WHO reclassified drugs for DR-TB, prioritizing BDQ as a group A drug for treatment (9). Based on findings from the two large clinical trials, TB-PRACTECAL (10) and ZeNix (11), the 2022 WHO treatment guidelines for DR-TB (12) even recommended a 6-month ultra-short-course regimen containing BDQ, known as BPaLM (bedaquiline, pretomanid, linezolid, and moxifloxacin), for treating RR-TB. The final results of TB-PRACTECAL were released in February 2024 (13), confirming the non-inferiority of BPaLM compared to the standard regimen.

However, in real-world settings, BDQ may need to be prolonged beyond 6 months in certain circumstances, such as when the number of effective drugs is insufficient to form an effective treatment regimen after 6 months of intensive therapy. This insufficiency may arise due to pre-extensively drug-resistant TB (pre-XDR-TB) strains, intolerable adverse drug reactions, lack of sputum culture conversion, or inadequate clinical response. There are only a few studies on the prolonged use of BDQ (7, 14, 15). While evidence supports the safe use of BDQ beyond 6 months for patients who receive appropriate baseline and follow-up monitoring, its use beyond this period remains off-label (12). The study by Letizia Trevisi et al. indicated that prolonging BDQ treatment did not enhance the treatment success rate (16). Thus, the effectiveness and safety data regarding prolonged BDQ treatment are still lacking, and the additional benefits of prolonging BDQ use remain unclear. Based on a real-world retrospective observational cohort study, this research compared the effectiveness and safety of the prolonged group with the 6-month group, balancing the baseline differences between the two groups through propensity score matching (PSM). After controlling for confounding factors, the effectiveness and safety of prolonged BDQ treatment for DR-TB were systematically evaluated.

## MATERIALS AND METHODS

### Study design and patients

This retrospective cohort study utilized real-world data. A total of 293 patients with DR-TB who received an anti-TB regimen containing BDQ were enrolled from three clinical centers between 1 March 2018 and 31 December 2021. Among them, 196 patients were from Shanghai Pulmonary Hospital affiliated to Tongji University, 68 patients were from the Third People’s Hospital of Zhenjiang affiliated to Jiangsu University, and 29 patients were from Shanghai Fengxian Guhua Hospital. Inclusion Criteria: 1. Age 16–80 years, regardless of gender; 2. HIV negative; 3. Diagnosed with DR-TB (including RR-TB, MDR-TB, and pre-XDR-TB), with BDQ included in the treatment regimen. Exclusion Criteria: 1. Patients who had not completed the course of treatment; 2. Patients for whom BDQ was used for less than 6 months; 3. Participation in other clinical trials; 4. Loss to follow-up or incomplete clinical data; 5. Interruption of BDQ treatment for more than 2 weeks.

The diagnostic criteria for DR-TB were based on WHO guidelines (12). RR-TB: TB disease caused by a strain of *Mycobacteria tuberculosis* (MTB) complex that is resistant to rifampicin. These strains may be susceptible or resistant to isoniazid. MDR-TB: TB disease caused by a strain of MTB complex that is resistant to both rifampicin and isoniazid. Pre-XDR-TB: TB disease caused by a strain of MTB complex that is resistant to rifampicin (and may also be resistant to isoniazid) and resistant to at least one fluoroquinolone (either levofloxacin or moxifloxacin).

The diagnostic process for DR-TB was based on the Companion Handbook to the WHO Guidelines for the Programmatic Management of DR-Tuberculosis (17) and updated according to the latest guideline (12). At the patient’s first visit, sputum or lavage fluid samples were sent for GeneXpert and liquid culture examinations. If GeneXpert indicated rifampicin resistance and the patient was classified as high-risk for DR-TB, RR-TB treatment was initiated immediately. Concurrently, isoniazid and second-line drug molecular resistance testing were performed while awaiting the results of traditional drug susceptibility testing (DST). The diagnosis and treatment regimen were adjusted based on DST results. If the patient was classified as low-risk for DR-TB, they underwent molecular resistance testing and waited for the traditional DST results. If either the molecular test or traditional DST indicated rifampicin resistance, RR-TB diagnosis was established, and treatment for DR-TB commenced immediately.

### Strain identification and DST

BACTEC MGIT 960 Culture Method (Bidi Company, USA): A volume of 0.5 mL of the treated sample was added to MGIT culture tubes containing 0.8 mL of OADC, following the manufacturer’s standard procedure, and incubated in the MGIT 960 instrument for 6 weeks. MPB64 antigen detection, p-nitrobenzoic acid (PNB), and thiophene-2-carboxylic acid hydrazide (TCH) medium growth tests were used to identify MTB strains from culture-positive clinical isolates. Minimum Inhibitory Concentration (MIC) DST: According to the Clinical and Laboratory Standards Institute (CLSI) M24-A2 guidelines, the broth microdilution method was employed to detect the MIC of 12 antibiotics for MTB strains. The cut-off values for the DST were determined based on the CLSI M24-A2 guidelines and the product instructions from Thermo Fisher’s tests.

GeneXpert MTB/RIF: The GeneXpert MTB/RIF testing was carried out following the manufacturer’s instructions (Cepheid GeneXpert System, Sunnyvale, CA, USA). Briefly, a 1 mL aliquot of the decontaminated specimen was mixed with 2 mL Xpert sample-processing reagent, vortexed for at least 10 s, and incubated at room temperature for 10 min. The mixture was then vortexed for another 10 s and incubated at room temperature for 5 min. A 2 mL aliquot of the mixture was transferred into the GeneXpert cartridge and loaded into the GeneXpert instrument, and the automatic detection procedure was initiated. The mutations in the rifampicin resistance determining region (RRDR) of the *rpoB* gene (codons 507–533) were detected.

### Anti-TB treatment and follow-up

The selection and dosage of drugs for DR-TB were based on the WHO guidelines for DR-TB treatment (12, 18) and were discussed by the Shanghai Expert Group for drug-resistance. BDQ was administered at a dose of 400 mg once daily with the morning meal during the initial 2 week loading phase, followed by a maintenance dose of 200 mg three times weekly (with intervals of at least 48 h, taken with food). If the corrected QT interval (QTc) exceeded 500 ms (confirmed by repeated electrocardiogram [ECG]), the drug should be suspended immediately (18). If the efficacy of the BDQ regimen proved unsatisfactory after 6 months of the intensive phase, or if patients could not tolerate the adverse reactions of other drugs, or if cultures failed to turn negative at the end of the intensive phase, or for other reasons (such as severe illness, pulmonary cavities, or extrapulmonary tuberculosis), the decision to prolong BDQ treatment was made based on the patient’s condition after discussion among the Shanghai Expert Group for drug-resistance. In cases of adverse drug reactions, the dose should be reduced or the drug should be discontinued in accordance with the guidelines (17). Any regimen adjustments required discussion and approval by the expert group.

The starting point was the initiation of anti-TB treatment containing BDQ for each patient, and the endpoints included the completion of treatment and discontinuation for various reasons. The observation period concluded 1 year after the end of treatment. Baseline levels were determined between 4 weeks before and 2 weeks after the initiation of anti-TB treatment, including demographic characteristics (gender, age, height, weight), clinical symptoms (cough, expectoration, hemoptysis, fever, dyspnea), complications (e.g., Diabetes), laboratory examinations, ECG, MTB molecular detection, MTB culture results, DST results, chest computed tomography (CT) examinations, and other relevant items. Patients were followed up monthly after the commencement of treatment, with continuous recording of clinical symptoms and adverse drug reactions (nausea, vomiting, loss of appetite, vision and hearing loss, etc.) during follow-up. Blood routine tests, liver and kidney function assessments, electrolyte levels, sputum smears, and cultures were reviewed monthly. ECGs were reviewed at 2, 12, and 24 weeks, and monthly thereafter 24 weeks of BDQ treatment. Chest CT scans were reexamined every 3 months (19) until the end of treatment.

### Treatment outcome

Treatment outcome were evaluated according to standard WHO definitions (20). Treatment failed: a patient whose treatment regimen needed to be terminated or permanently changed^a^ (at least two anti-TB drugs) to a new regimen or treatment strategy. Treatment completed: a patient who completed treatment as recommended by the national policy, whose outcome does not meet the definition for cure or treatment failure. Cured: a pulmonary TB patient with bacteriologically confirmed TB at the beginning of treatment who completed treatment as recommended by the national policy, with evidence of bacteriological response^b^ and no evidence of failure. Died: a patient who died before or during the course of treatment. Lost to follow-up: a patient who did not start treatment or whose treatment was interrupted for two consecutive months or more. Treatment success: the sum of cured and treatment completed.

^a^ Reasons for the change include：no clinical response and/or no bacteriological response (see note “b”); adverse drug reactions; or evidence of additional drug resistance to medicines in the regimen.

^b^ Bacteriological conversion: a situation in a patient with bacteriologically confirmed TB where at least two consecutive cultures taken on different occasions at least 7 days apart are negative.

### Statistical analysis

Data were processed using SPSS version 23.0 software (IBM SPSS Statistics, Chicago, IL, USA). Non-normally distributed continuous variables were expressed as interquartile ranges (IQR) and compared using the *Mann-Whitney U* test. Categorical variables were expressed as frequencies (proportions %) and compared with the Chi-square test or Fisher’s exact test (when the total sample size was less than 40 or the theoretical frequency was less than 5). Kaplan-Meier curves were generated using GraphPad Prism 9 (GraphPad Software, LLC) to compare culture conversion times and pulmonary cavity closure times between the two groups, with the log-rank test used to determine the statistical significance of differences. A two-sided test with *P* the statistical significance of differences. A two-sided test with *P* ≤ 0.05 was considered statistically significant.

The PSM was employed to analyze the effect of prolonged BDQ treatment on outcomes. PSM utilized the nearest neighbor algorithm with 1:1 matching, a caliper value set at 0.1 (to reduce sample size loss while ensuring matching balance), and a non-repeated matching method. The variables for PSM were determined based on the comparison of baseline levels of BDQ treatment duration (6-month group and prolonged group) (Table 1), correlation analysis of factors affecting treatment outcomes (logistic regression, results shown in Table S1), and clinical experience (Table S2). Standard deviation analysis was performed on the matched samples; a standard deviation of less than 0.2 indicated balanced matching (see Table S2). The comparison of baseline levels after matching is presented in Table S3. Additionally, we stratified the BDQ prolonged groups (7–9 months, 10–12 months, and >12 months) and compared baseline levels before and after PSM adjustment (see Tables S4, S7, and S10). Influencing factors on treatment outcomes were analyzed (see Tables S5, S8, and S11 for logistic regression results). The matching balance results are shown in Tables S6, S9, and S12, respectively. For those patients in the 6-month group who met the requirements for BDQ prolongation but did not prolong, Chi-square tests were performed on the treatment outcomes with the prolonged group before and after PSM (baseline comparison is shown in Table S13, logistic regression results are shown in Table S14, and matching balance results are shown in Table S15). The propensity score was implemented using the R Programming Language (Copyright (C) 2009–2024 by Posit Software, PBC, version number: 2023.12.01 Build 402).

**TABLE 1:** Baseline demographics and characteristics^*a*^

Variable	Number (%)		*P*
	6 mo (*n* = 72)	>6 mo (*n* = 88)	
Age			
<18 y	4 (5.6)	3 (3.4)	0.509
18–35 y	33 (45.8)	47 (53.4)	0.340
35–45 y	16 (22.2)	15 (17.0)	0.410
45–60 y	10 (13.9)	18 (20.5)	0.277
≥60 y	9 (12.5)	5 (5.7)	0.129
Sex			
Male	48 (66.7)	59 (67.0)	0.960
Female	24 (33.3)	29 (33.0)	
BMI (kg/m^2^)			
<18.5	19 (26.4)	15 (18.7)	0.151
18.5–25	49 (68.1)	66 (75.0)	0.331
≥25	4 (5.6)	7 (8.0)	0.755
Diabetes	9 (12.5)	11 (12.5)	1.0
Resistance category			
RR-TB	2 (2.8)	11 (12.5)	0.025
MDR-TB	17 (23.6)	29 (33.0)	0.194
pre-XDR-TB	53 (73.6)	48 (54.5)	0.013
Patient treatment category			
New	25 (34.7)	23 (26.1)	0.238
Retreatment	47 (65.3)	65 (73.9)	
Bronchial tuberculosis	12 (16.7)	12 (13.6)	0.593
Tuberculous pleurisy	3 (4.2)	11 (12.5)	0.063
Extrapulmonary tuberculosis	1 (1.4)	2 (2.3)	1.0
Microbiologic culture at baseline			
Positive	68 (94.4)	78 (88.6)	0.196
Imaging (CT)			
Scope of lesions (lobes)			
1–2	29 (40.3)	39 (44.3)	0.607
3–4	18 (25.0)	17 (20.5)	0.493
5–6	25 (34.7)	31 (35.2)	0.947
Cavity	30 (41.7)	48 (54.5)	0.105
Clinical symptoms			
Cough	46 (63.9)	48 (54.5)	0.232
Expectoration	40 (55.6)	42 (46.6)	0.259
Fever	12 (16.7)	15 (17.0)	0.949
Hemoptysis	17 (23.6)	14 (15.9)	0.220
Dyspnea	6 (8.3)	11 (12.5)	0.395

^*a*^ Data are *n* (%). BMI, body mass index; RR-TB, rifampicin-resistant tuberculosis; MDR, multidrug-resistant tuberculosis; pre-XDR, pre-extensively drug-resistant tuberculosis; mo, months; CT, computed tomography. Percentages calculated based on the number of patients with a result at baseline (baseline refers to 4 weeks before or 2 weeks after treatment initiation).

## RESULTS

### Baseline comparison between the prolonged group and the 6-month group

**Fig 1:**
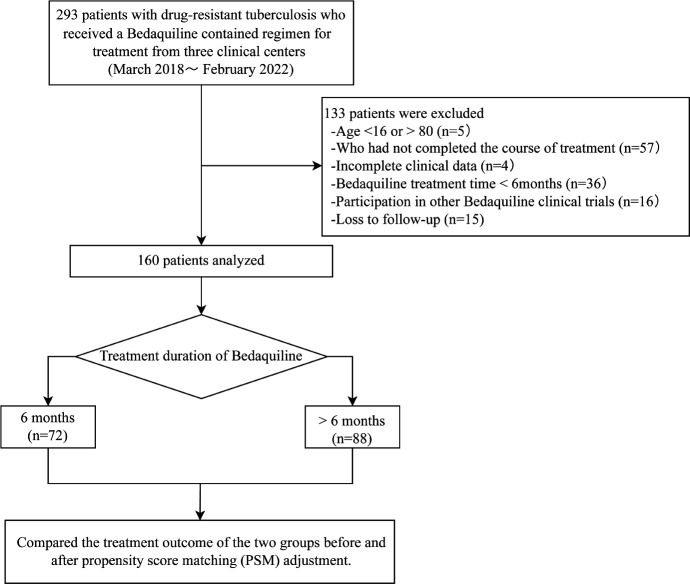
Flowchart of analysis inclusion. Abbreviations: TB, tuberculosis; PSM, propensity score matching.

From March 2018 to December 2021, a total of 293 patients with DR-TB who received an anti-TB regimen containing BDQ were enrolled. Ultimately, 160 patients who met the criteria were included in the analysis (see Fig. 1). Among them, 72 patients received BDQ treatment for 6 months (24 weeks), and 88 patients received BDQ treatment for more than 6 months. The comparison of baseline characteristics between the two groups was shown in Table 1. Significant differences were observed in the types of DR-TB between the two groups. The proportion of RR-TB in the prolonged group was higher than the 6-month group (12.5% vs 2.8%, *P* = 0.025), while the proportion of pre-XDR-TB in the prolonged group was lower than the 6-month group (54.5% vs 73.6%, *P* = 0.013). No significant differences were found in age, gender, body mass index (BMI), baseline clinical symptoms, type of TB treatment (initial treatment vs retreatment), comorbidities (Diabetes), TB infection site, imaging findings, and the positive rate of sputum culture between the two groups (all *P* > 0.05).

### Phenotypic DST results and frequency of Anti-TB drugs used between the two groups

As presented in Table 2, the phenotypic DST results revealed no statistically significant differences in resistance rates for most antibiotics, with the exception of kanamycin (*P* = 0.015). There were no statistically significant differences in the use of fluoroquinolones (levofloxacin and moxifloxacin), linezolid, clofazimine, cycloserine, ethambutol, delamanid, pyrazinamide, injectable drugs (Amikacin/Capreomycin), prothionamide, and sodium p-aminosalicylic acid (all *P* > 0.05, Table 3). In the prolonged group, the median duration of BDQ use was 9 months (IQR: 8–11 months), with 50 cases (56.8%) receiving BDQ for 7–9 months, 21 cases (23.9%) for 10–12 months, and 17 cases (19.3%) for more than 12 months.

**TABLE 2:** Phenotypic drug susceptibility testing of *Mycobacteria tuberculosis* isolates^*a*^

Drug	Resistant number (%)		*Χ* ^2^	*P*
	6 mo (*n* = 72)	>6 mo (*n* = 88)		
Isoniazid	66 (91.7)	75 (85.2)	1.569	0.210
Rifampicin	67 (93.1)	75 (85.2)	2.431	0.119
Rifabutin	56 (77.8)	59 (67.0)	2.256	0.133
Ethambutol	39 (54.2)	38 (43.2)	1.914	0.167
Fluoroquinolone	51 (70.8)	54 (61.4)	1.574	0.210
Amikacin	20 (27.8)	14 (15.9)	3.333	0.068
Streptomycin	50 (69.4)	54 (61.4)	1.137	0.286
Kanamycin	20 (27.8)	11 (12.5)	5.917	**0.015**
Ethylthioisonicotinamide	16 (22.2)	22 (25.0)	0.169	0.681
Cycloserine	12 (16.7)	8 (9.1)	2.078	0.149
Sodium p-aminosalicylate	10 (13.9)	12 (13.6)	0.002	0.963

^*a*^ Data are *n* (%). mo, months.

**TABLE 3:** Frequency of anti-tuberculosis drugs used between the two groups^*a*^

Variable	Number (%)		*P*
	6 mo (*n* = 72)	>6 mo (*n* = 88)	
Fluoroquinolone	14 (22.4)	21 (25)	0.501
Linezolid	65 (90.3)	77 (87.5)	0.580
Clofazimine	55 (76.4%)	58 (65.9)	0.148
Cycloserine	54 (75.0)	68 (77.3)	0.737
Ethambutol	6 (8.3)	6 (6.8)	0.717
Delamanid	2 (2.8)	3 (3.4)	0.819
Pyrazinamide	36 (50.0)	46 (52.3)	0.775
Amikacin/Capreomycin	30 (41.7)	30 (34.1)	0.325
Prothionamide	45 (62.5)	48 (54.5)	0.310
Sodium p-aminosalicylate	27 (37.5)	26 (29.5)	0.288

^*a*^ Data are *n* (%). mo, months.

### Treatment outcome

The cure rate, treatment success rate, and treatment failure rate in the prolonged group did not significantly differ from those in the 6-month group (55.7% vs 52.8%, 78.4% vs 77.8%, and 19.3% vs 22.2%, all *P* > 0.05). There were two deaths in the prolonged group and none in the 6-month group (Table 4).

**TABLE 4:** Treatment outcomes^*a*^

Outcomes	Number (%)		*P*
	6 mo (*n* = 72)	>6 mo (*n* = 88)	
Cured	38 (52.8)	49 (55.7)	0.751
Treatment completed	18 (25.0)	20 (22.7)	0.737
Treatment success	56 (77.8)	69 (78.4)	0.923
Failed	16 (22.2)	17 (19.3)	0.652
Death	0 (0.0)	2 (2.3)	0.502
Sputum culture conversion	66 (97.1)	70 (94.6)	0.682
Imaging evaluation (CT)			
Improvement	63 (87.5)	71 (80.7)	0.172
Unchanged	6 (8.3)	13 (14.8)	0.210
Progression	3 (4.2)	4 (4.5)	1.0
Cavitary evaluation			
Closed	14 (46.7)	23 (47.9)	0.914
Smaller	12 (40.0)	16 (33.3)	0.550
Unchanged	3 (10.0)	5 (10.4)	1.0
Progression	1 (3.3)	4 (8.3)	0.644
Symptom improvement			
Cough	44 (95.7)	47 (97.9)	0.613
Expectoration	38 (95.0)	40 (97.6)	0.616
Fever	12 (100)	13 (86.7)	0.487
Hemoptysis	15 (88.2)	13 (92.9)	1.0
Dyspnea	6 (100.0)	8 (72.7)	0.515

^*a*^ Data are *n* (%). CT, computed tomography; mo, months.

**Fig 2:**
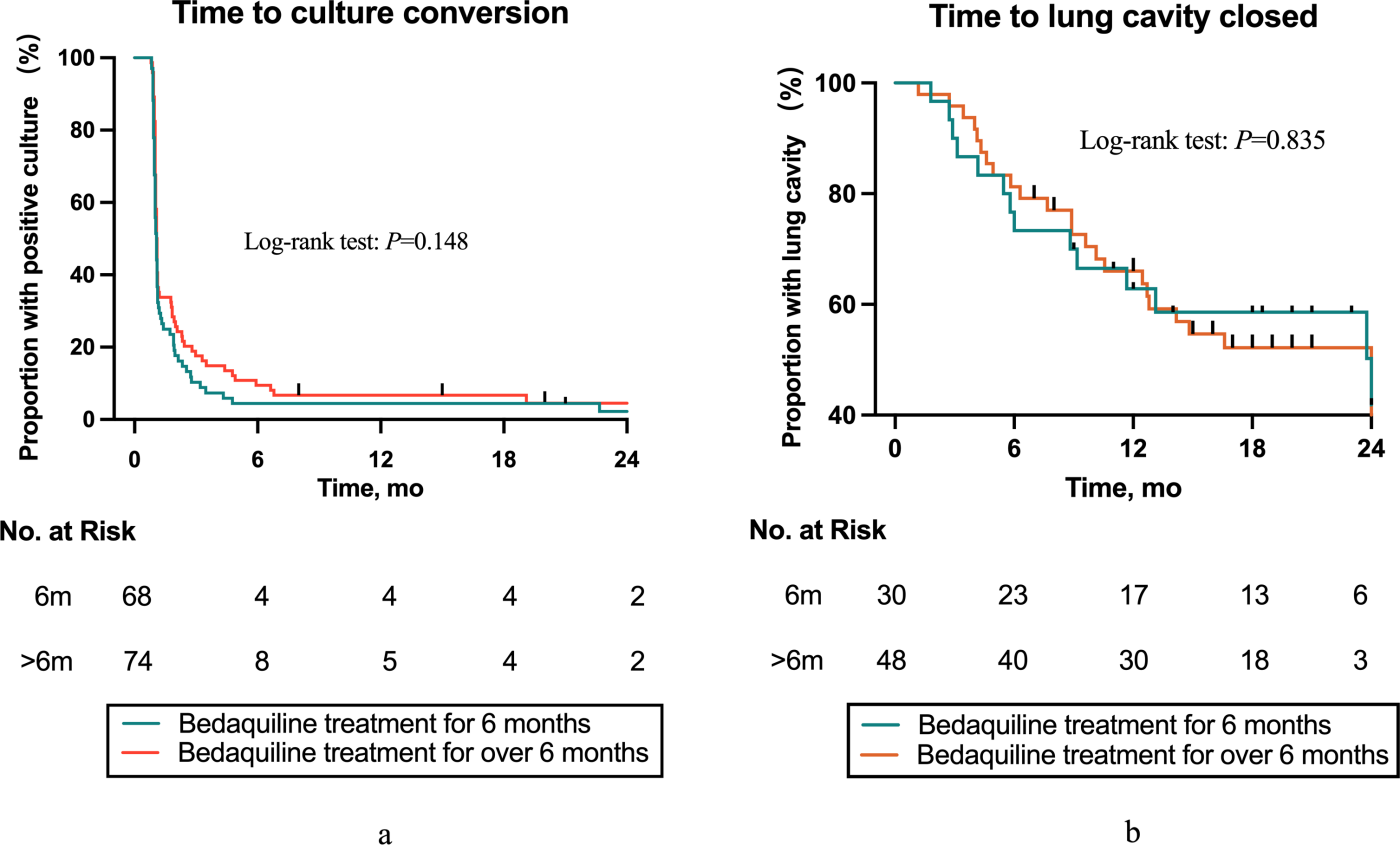
(**a**) Kaplan-Meier graph showing the time to initial sputum culture conversion in drug-resistant TB patients with positive sputum cultures. (**b**) Kaplan-Meier graph showing the time to initial cavity closure in drug-resistant TB patients with pulmonary cavity.

The sputum culture conversion rates for the prolonged group and the 6-month group were 94.6% and 97.1%, respectively (*P* = 0.682, Table 4). The median times to culture conversion were 32 days (IQR: 30–55 days) and 31.5 days (IQR: 29–39.5 days), respectively, with no statistically significant difference (*P* = 0.160). No sputum culture recurrences occurred in either group. The results of the Kaplan-Meier curve indicated that prolonging BDQ did not significantly increase the rate of sputum culture conversion (*P* = 0.148, Fig. 2a).

No statistically significant differences were observed in radiographic outcomes and cavity closure rates between the prolonged group and the 6-month group (Table 4). The median time to cavity closure in the two groups were 8.9 months (IQR: 4.47–12.58 months) and 5.9 months (IQR: 3.39–11.04 months), respectively (*P* = 0.526). Kaplan-Meier curves showed that the prolongation of BDQ did not significantly increase the rate of cavity closure (*P* = 0.835, Fig. 2b).

Clinical symptoms in both groups improved greatly after treatment, with cough, expectoration, and fever symptoms showing improvements of over 90%. However, these differences were not statistically significant (all *P* > 0.05, Table 4).

### Treatment outcomes after PSM

**Fig 3:**
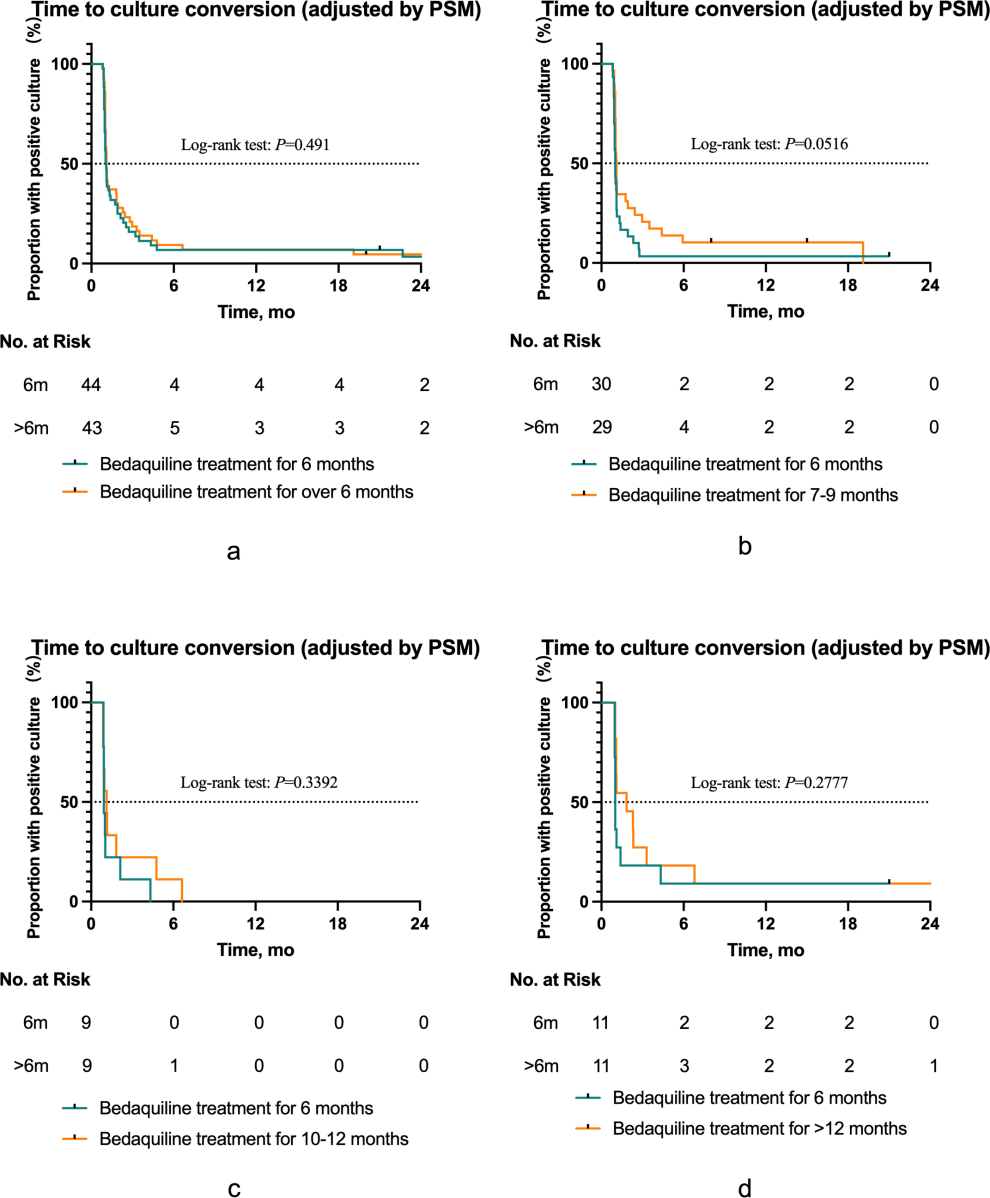
(**a through d**) Kaplan-Meier graphs comparing the time to initial sputum culture conversion between the 6-month group and stratified prolonged groups after adjustment by propensity score matching (PSM).

In this study, PSM was employed to adjust for baseline levels and evaluate the treatment success rate of the BDQ prolonged group vs the 6-month group (see Table 5). After baseline adjustment, there was no significant difference in treatment success rates between the prolonged group and the 6-month group (SR = 0.97, 95% CI: 0.76–1.24, *P* = 0.815). Additionally, there was no significant difference in the Kaplan-Meier curve for sputum culture conversion rates between the prolonged group and the 6-month group (*P* = 0.491, Fig. 3a). Stratified analysis of the prolonged groups revealed that there were no significant differences in treatment success rates compared between the 6-month group with the 7–9 months, 10–12 months, and >12 months groups (SR_7–9 mo_ = 1.04, 95% CI: 0.81–1.33; SR_10–12 mo_ = 0.88, 95% CI: 0.53–1.46; SR_>12 mo_ =0.88, 95% CI: 0.49–1.55; all *P* > 0.05) (Table 5). Furthermore, no significant differences were observed in the sputum culture conversion curves among the prolonged groups compared to the 6-month group (*P*_7–9 mo_ = 0.0516, *P*_10–12 mo_ = 0.3392, *P*_>12 mo_ =0.2777; Fig. 3b through d).

**TABLE 5:** Treatment success ratio before and after adjustment by propensity score matching (PSM)

Treatment duration of bedaquiline	SR^*a*^(95% CI)	*P*	SR^*a*^(95% CI)	*P*
	Unadjusted		Adjusted^*b*^	
6 mo (*n* = 72)	Ref.		Ref.	
Prolonged (*n* = 88)	1.01 (0.85–1.19)	0.923	0.97 (0.76–1.24) (*n* = 47)	0.815
7–9 mo (*n* = 50)	0.95 (0.79–1.13)	0.570	1.04 (0.81–1.33) (*n* = 32)	0.756
10–12 mo (*n* = 21)	1.22 (1.04–1.43)	0.586	0.88 (0.53–1.46) (*n* = 10)	1.000
≥12 mo (*n* = 17)	0.75 (0.499–1.15)	0.108	0.88 (0.49–1.55) (*n* = 11)	1.000

^*a*^ SR, success ratio.

^*b*^ The model was matched through propensity score matching, adjusted for age (subgroups), gender, BMI (kg/m^2^, subgroups), resistance category (RR-TB, MDR-TB, pre-XDR-TB), and other factors that were incompatible or affect treatment outcomes.

**Fig 4:**
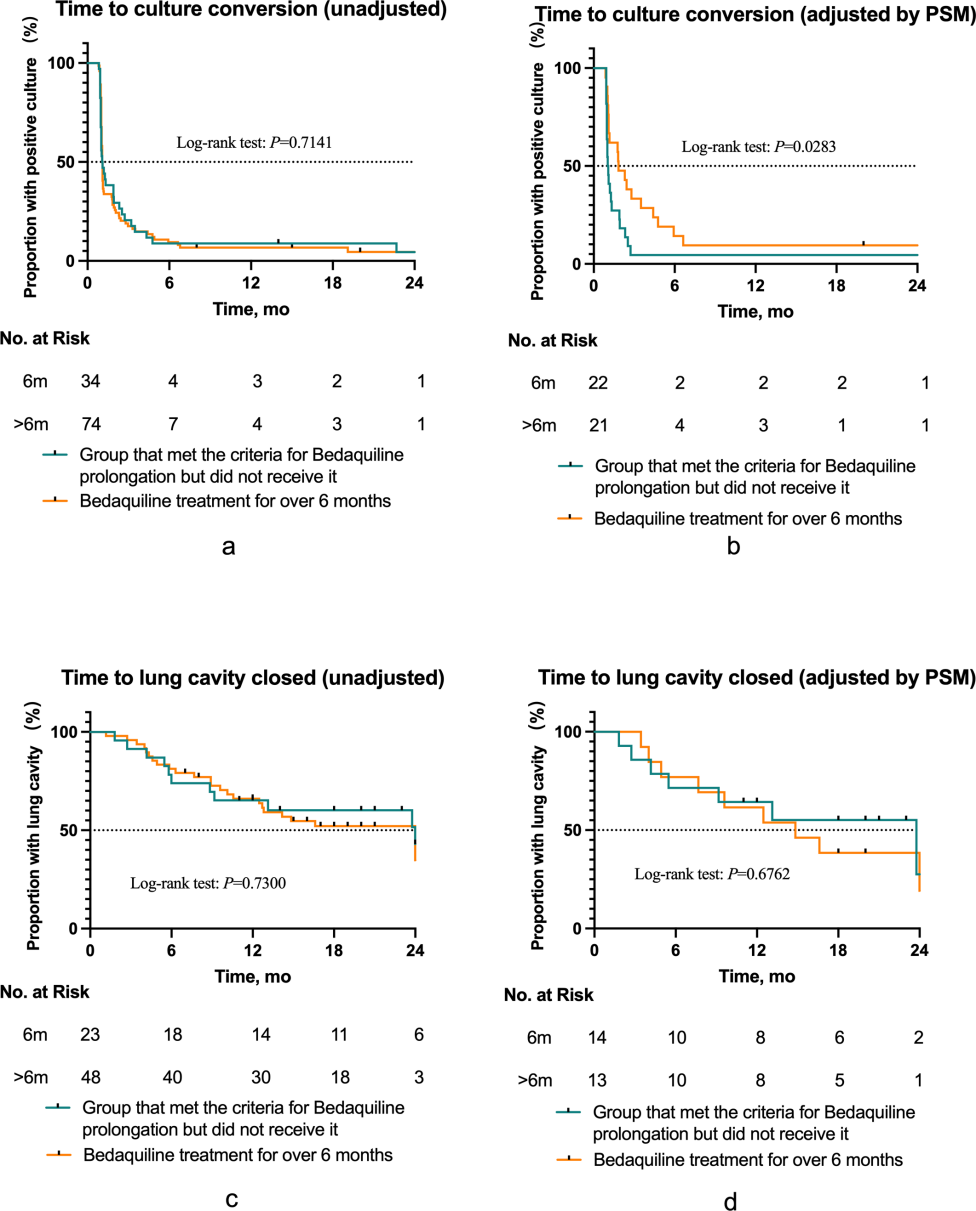
(**a, b**) Kaplan-Meier graphs comparing the time to initial sputum culture conversion between the prolonged group and the group that met the criteria for BDQ prolongation but did not receive it, before and after adjustment by propensity score matching (PSM). (**c, d**) Kaplan-Meier graphs comparing the time to initial cavity closure in patients with a baseline cavity between the prolonged group and the group that met the criteria for BDQ prolongation but did not receive it, before and after adjustment by PSM.

In the 6-month group, 35 patients met the criteria for BDQ prolongation but did not receive it, 2 patients due to QTc > 500 ms, 6 due to liver damage, and 27 due to economic reasons. The treatment success rate of the prolonged group (78.4%) was significantly higher than the non-prolonged group (60%, *P* = 0.038). The median treatment duration in the prolonged group (18 months, IQR: 15.00–20.25 months) was significantly shorter than that of the non-prolonged group (23 months, IQR: 18.50–25.00 months, *P* < 0.001; Table S13). However, no significant difference in treatment success rates was observed between the two groups after PSM (*P* = 0.768). The sputum culture conversion and cavity closure curves between the two groups also showed no significant differences (Fig. 4a through d).

### Adverse events

The comparison of common adverse reactions is shown in Table 6. The most prevalent adverse reaction was QT interval prolongation (44.4% in the 6-month group and 40.9% in the prolonged group, *P* = 0.653), followed by liver and kidney damage, peripheral neuropathy, and cytopenia. Drug fever, rash, and ototoxicity were relatively rare, with no significant differences observed between the two groups (all *P* > 0.05). Two patients in the 6-month group suspended BDQ due to QTc exceeding 500 ms (the duration of drug withdrawal was less than 2 weeks). Notably, two patients in the prolonged group died during treatment, while none in the 6-month group did.

**TABLE 6:** Adverse events (AEs)^*a*^

Variable	Number (%)		*P*
	6 mo (*n* = 72)	>6 mo (*n* = 88)	
QTc prolongation	32 (44.4)	36 (40.9)	0.653
QTc (ms)			
<450	40 (55.6)	52 (59.1)	0.653
450–500	30 (41.7)	36 (40.9)	0.923
≥500	2 (2.8)	0 (0.0)	0.201
Liver and kidney impairment	15 (20.8)	22 (25.0)	0.534
Hemogram reduction	11 (15.3)	11 (12.5)	0.612
Rash	1 (1.4)	3 (3.4)	0.628
Drug fever	1 (1.4)	1 (1.1)	1.0
Ototoxicity	1 (1.4)	6 (6.8)	0.13
Peripheral neuropathy	12 (16.7)	10 (11.4)	0.333
Blurred vision	4 (5.6)	13 (14.8)	0.06
Gastrointestinal adverse reaction	8 (11.1)	6 (6.8)	0.339
Mental	8 (11.1)	8 (9.1)	0.672
Death	0 (0%)	2 (2.3%)	0.502

^*a*^ Data are *n* (%). mo, months; QTc, corrected QT interval; ms, millisecond.

## DISCUSSION

In this real-world retrospective observational cohort study, no significant differences were observed in age, sex, BMI, clinical symptoms, previous anti-TB treatment, diabetes, TB location, imaging findings (lesion size and cavity condition), and background drug selection among patients at baseline. The proportion of RR-TB in the prolonged group was higher than that in the 6-month group. A review of the clinical data revealed that 7 of the 11 RR-TB patients in the prolonged group had negative MTB cultures, with the diagnosis relying solely on GeneXpert results. The DST results for other anti-TB drugs were unknown. These patients experienced poor efficacy or adverse reactions to other drugs, leading to a prolonged BDQ treatment course. Our results demonstrated no significant association between treatment outcomes and resistance categories (Table S1), and there was no statistically significant difference in treatment success rates between the two groups, both before and after PSM adjustment for baseline characteristics. The selection of therapeutic drugs adhered to the recommended order of groups A, B, and C as per WHO guidelines (12). China has a high resistance rate to fluoroquinolones, with the fluoroquinolone resistance rate among the patients included in this study at 67.5% (Table 2). Consequently, the use of fluoroquinolones was less frequent than that of some group C drugs. The high proportion of injectable drugs stemmed from the fact that these medications remained mainstays for the long-term treatment of RR-TB prior to the 2018 update of the WHO drug resistance TB treatment guidelines (21).

The results of this study indicated that the treatment success rate of the BDQ prolonged group was similar to that of the 6-month group and slightly higher than that reported by WHO (1). Using the PSM method, we divided the prolonged group into 7–9 months, 10–12 months, and over 12 months. Compared to the 6-month group, there were no significant differences in treatment success rates, sputum culture conversion rates, and cavity closure rates between the prolonged and 6-month groups. However, our results showed that the treatment success rate in the prolonged group was significantly higher than that of the group met the criteria for BDQ prolongation but did not receive it. Although no significant difference in treatment success rates was observed between the two groups after PSM, the median treatment duration in the prolonged group remained shorter. As a result, prolonged use of BDQ can achieve similar or improved treatment outcomes while potentially shortening the overall anti-TB course, ultimately benefiting the patients. Lorenzo Guglielmetti et al. found that BDQ prolongation treatment was generally well tolerated, yet no significant difference in treatment outcomes was noted between the standard and prolonged groups (22). Several studies also suggested no difference in treatment success rates between the prolonged and standard groups (16, 23), which is consistent with our findings. Additionally, some experts recommend the long-term use of BDQ for treating highly DR-MTB strains (24). Therefore, it is advisable to prolong BDQ treatment for patients who cannot establish an effective treatment regimen after 6 months of the intensive phase, those who are intolerant to other drugs due to adverse reactions, or those whose cultures fail to turn negative by the end of the intensive phase, as well as for other reasons including severe illness, pulmonary cavities, or extrapulmonary tuberculosis.

In this study, the treatment success rate was 77.8% in the 6-month group and 78.4% in the prolonged group, while the sputum culture conversion rates were 97.1% and 94.6%, respectively. This discrepancy may be attributed to the WHO definition of treatment failure, which includes permanent adjustments of at least two anti-TB drugs. Following adjustments to treatment regimens,some patients were still able to achieve clinical success.

Adverse drug reactions pose a significant challenge in the treatment of DR-TB and may even impact the final treatment outcome. A meta-analysis involving 35 studies and 9,178 patients (25) indicated that fluoroquinolones, clofazimine, and BDQ had the lowest incidence of adverse events leading to permanent withdrawal, whereas second-line injectable drugs, para-aminosalicylic acid, and linezolid exhibited the highest rates of discontinuation. Common adverse reactions associated with BDQ include gastrointestinal symptoms (nausea, vomiting, abdominal pain, and loss of appetite), joint pain, headache, and QTc interval prolongation, a particular clinical concern (17). In this study, no significant differences were observed in the incidence of adverse drug reactions between the 6-month group and the prolonged group. In the prolonged group, 7 out of 17 patients treated with BDQ, moxifloxacin, and clofazimine experienced QTc prolongation; however, none of these patients discontinued BDQ under close observation.

It is noteworthy that two patients in the prolonged group died during treatment in this study. Case 1 involved a 27-year-old man with a low body weight (42 kg) and a BMI of 14 kg/m². He had undergone right lung lobectomy and interventional therapy for hemoptysis and was complicated by pulmonary aspergillosis. He unfortunately died of acute cholecystitis and acute abdomen. There was no evidence to indicate that the fatal outcome was associated with anti-TB treatment. Case 2 was a 73-year-old male with poorly controlled diabetes mellitus. A review of his history revealed baseline QTc prolongation (460 ms), with intermittent QTc prolongation (QTc between 450 ms and 500 ms). Considering the patient had pre-XDR-TB with widespread lesions (involving five lung lobes and multiple cavities), and his sputum culture did not turn negative, he was advised to continue BDQ with close follow-up. Unfortunately, he died suddenly 10 months after starting the BDQ-containing treatment. The anti-TB treatment could not be ruled out as a direct cause of death. This patient exhibited poor compliance and irregular follow-up, and his ECG was not strictly rechecked monthly after 6 months of BDQ use, impeding timely identification and intervention for potential risks. This underscores the importance of strict regular follow-up when prolonging BDQ use. BDQ is not recommended for patients who cannot adhere to timely follow-up and have poor compliance. The results of a prospective cohort study examining the efficacy and cardiac safety of BDQ in treating DR-TB (26) suggest that severe QTc prolongation is uncommon and does not necessitate the permanent discontinuation of BDQ or clofazimine. Therefore, we believe that prolonging BDQ treatment, when accompanied by good patient compliance and close monitoring, is safe and recommended (15, 22, 27).

Although the effective half-life of BDQ is approximately 24–30 h, its terminal half-life is about 4–5 months, blood concentrations of BDQ can still be measured 200 days after discontinuation (28). BDQ treatment was associated with an increase in MIC of BDQ and the emergence of *mmpR* and *atpE* gene mutations in MTB strains (29). Furthermore, it was found that the initial BDQ resistance rate among MDR patients in China was low, with an acquired resistance rate of 2.2% (5/232) (30). Whether prolonging BDQ treatment will lead to increased resistance remains unknown. Therefore, as one of the most effective anti-TB drugs currently available, BDQ should be used with caution when prolonged. At our centers, any prolongation of BDQ must be discussed and approved by the Shanghai Expert Group for drug-resistance.

This study has certain limitations. Although we collected clinical data in a real-world setting from three clinical centers and utilized PSM to adjust for baseline characteristics of all enrolled patients, the study design was retrospective, and the study population was not large. Therefore, the efficacy and safety of prolonged BDQ use still require further validation through prospective, randomized controlled clinical trials.

### Conclusion

This study found no significant difference in effectiveness and safety between the prolonged and the 6-month groups among DR-TB patients. Prolonged use of BDQ achieved similar treatment outcomes while potentially shortening the overall anti-TB treatment duration. Therefore, when available anti-TB drugs are insufficient to establish an effective treatment regimen, it is recommended to prolong BDQ use under close monitoring. Due to the cardiotoxicity associated with BDQ, it is essential to closely monitor ECG and QTc intervals during treatment. Prolonged use of BDQ is not recommended for patients who cannot adhere to timely follow-up and demonstrate poor compliance.

## Data Availability

The data used during the current study are available from the corresponding author upon reasonable request.

## SUPPLEMENTAL MATERIAL

Supplemental tables spectrum.00097-25-s0001.docx Tables S1 to S15.

ASM does not own the copyrights to Supplemental Material that may be linked to, or accessed through, an article. The authors have granted ASM a non-exclusive, world-wide license to publish the Supplemental Material files. Please contact the corresponding author directly for reuse.
