# Supplementary material for: Real-world effectiveness and safety of prolonged bedaquiline course in the treatment of drug-resistant tuberculosis—a multi-center retrospective cohort study in a country with a high burden of drug-resistant tuberculosis
Source: Microbiol Spectr. 2025 Jul 7;13(8):e00097-25. doi: 10.1128/spectrum.00097-25 (PMC12323657; doi:10.1128/spectrum.00097-25)
Supplement: Supplemental tables — Tables S1 to S15. [file spectrum.00097-25-s0001.docx]

**Table S1. Correlation analysis of factors affecting treatment outcomes (6-month and prolonged groups)**

| Dependent: Outcome | Success  (n=125) | Failure  (n=35) | OR (univariable)  (95% CI，*P*） |
| --- | --- | --- | --- |
| **Gender** | | | |
| Male | 84 (67.2) | 23 (65.7) |  |
| Female | 41 (32.8) | 12 (34.3) | 1.07 (0.48-2.36, *P*=0.869) |
| **Age** | | | |
| <18y | 6 (4.8) | 1 (2.9%) |  |
| 18-35y | 63 (50.4) | 17 (48.6) | 1.62 (0.18-14.38, *P=*0.665) |
| 35-45y | 23 (18.4) | 8 (22.9) | 2.09 (0.22-20.09, *P=*0.524) |
| 45-60y | 22 (17.6) | 6 (17.1) | 1.64 (0.16-16.34, *P=*0.675) |
| ≥60y | 11 (8.8) | 3 (8.6) | 1.64 (0.14-19.39, *P=*0.696) |
| **BMI (kg/m^2^)** | | | |
| <18.5 | 24 (19.2) | 10 (28.6) |  |
| 18.5-25 | 91 (72.8) | 24 (68.6) | 0.63 (0.27-1.50, *P=*0.299) |
| ≥25 | 10 (8) | 1 (2.9) | 0.24 (0.03-2.13, *P=*0.200) |
| **Resistance category** | | | |
| RR-TB | 11 (8.8) | 2 (5.7) |  |
| MDR-TB | 39 (31.2) | 7 (20.0) | 0.99 (0.18-5.45, *P*=0.988) |
| pre-XDR-TB | 75 (60.0) | 26 (74.3) | 1.91 (0.40-9.18, *P*=0.421) |
| **Treatment times (mo), Mean ± SD** | 18.0 ± 4.9 | 21.7 ± 7.5 | **1.11 (1.04-1.19, *P*=0.002)** |
| **Bronchial tuberculosis** | 18 (14.4) | 6 (17.1) | 1.23 (0.45-3.38, *P*=0.688) |
| **Tuberculous pleurisy** | 9 (7.2) | 5 (14.3) | 2.15 (0.67-6.88, *P*=0.198) |
| **Extrapulmonary** | 4 (3.2) | 1 (2.9) | 0.89 (0.10-8.23, *P*=0.918) |
| **Treatment category** | | | |
| New | 37 (29.6) | 11 (31.4) |  |
| Retreatment | 88 (70.4) | 24 (68.6) | 0.92 (0.41-2.06, *P*=0.835) |
| **Diabetes** | 14 (11.2) | 6 (17.1) | 1.64 (0.58-4.64, *P*=0.351) |
| **Culture at baseline (Positive)** | 114 (91.2) | 32 (91.4) | 1.03 (0.27-3.91, *P*=0.966) |
| **Clinical symptoms** |  |  |  |
| Cough | 73 (58.4) | 21 (60) | 1.07 (0.50-2.29, *P*=0.865) |
| Expectoration | 63 (50.4) | 18 (51.4) | 1.04 (0.49-2.21, *P*=0.914) |
| Fever | 22 (17.6) | 5 (14.3) | 0.78 (0.27-2.24, *P*=0.644) |
| Hemoptysis | 24 (19.2) | 7 (20.0) | 1.05 (0.41-2.69, *P=*0.916) |
| Dyspnea | 13 (10.4) | 4 (11.4) | 1.11 (0.34-3.65, *P=*0.862) |
| **Cavities** | 54 (43.2) | 24 (68.6) | **2.87 (1.29-6.36, *P*=0.009)** |
| **Scope of lesions (lobes)** | | | |
| 1-2 | 58 (46.4) | 10 (28.6) |  |
| 3-4 | 26 (20.8) | 10 (28.6) | 2.23 (0.83-6.01, *P*=0.113) |
| 5-6 | 41 (32.8) | 15 (42.9) | 2.12 (0.87-5.19, *P*=0.099**)** |
| **Drugs** |  |  |  |
| Linezolid | 110 (88) | 32 (91.4) | 1.45 (0.40-5.34, *P*=0.572) |
| Fluoroquinolone | 27 (21.6) | 8 (22.9) | 1.08 (0.44-2.64, *P*=0.874) |
| Clofazimine | 86 (68.8) | 27 (77.1) | 1.53 (0.64-3.67, *P*=0.340) |
| Cycloserine | 100 (80.0) | 22 (62.9) | **0.42 (0.19-0.95, *P*=0.038)** |
| Pyrazinamide | 66 (52.8) | 16 (45.7) | 0.75 (0.35-1.60, *P*=0.459) |
| Ethambutol | 7 (5.6) | 5 (14.3) | 2.81 (0.83-9.47, *P*=0.096) |
| Delamanid | 5 (4.0) | 0 (0.0) | 0.00 (0.00-Inf, *P*=0.989) |
| Amikacin/Capreomycin | 40 (32.0) | 20 (57.1) | **2.83 (1.31-6.11, *P*=0.008)** |
| Sodium p-aminosalicylate | 42 (33.6) | 11 (31.4) | 0.91 (0.41-2.02, *P*=0.809) |
| Prothionamide | 74 (59.2) | 19 (54.3) | 0.82 (0.38-1.74, *P*=0.603) |
| Data are n (%) or Mean **±** SD. SD: standard deviation, OR: odds ratio, mo: month, BMI: body mass index, RR-TB: rifampicin-resistant tuberculosis, MDR: multidrug-resistant tuberculosis, pre-XDR: pre-extensively drug-resistant tuberculosis. Percentages calculated based on the number of patients with a result at baseline (baseline refers to 4 weeks before or 2 weeks after treatment initiation). | | | |

**Table S2. Balance test of PSM (6-month and prolonged groups)**

|  | Type | Diff.Un | Diff.Adj | M.Threshold |
| --- | --- | --- | --- | --- |
| distance | Distance | 0.9451 | 0.0473 |  |
| **Gender** | Binary | -0.0038 | -0.0213 | Balanced, <0.2 |
| **Age** | | | | |
| <18y | Binary | -0.0215 | -0.0426 | Balanced, <0.2 |
| 18-35y | Binary | 0.0758 | 0.0213 | Balanced, <0.2 |
| 35-45 | Binary | -0.0518 | 0.0000 | Balanced, <0.2 |
| 45-60 | Binary | 0.0657 | 0.0000 | Balanced, <0.2 |
| ≥60y | Binary | -0.0682 | 0.0213 | Balanced, <0.2 |
| **BMI (kg/m^2^)** | | | | |
| <18.5 | Binary | -0.0934 | 0.0213 | Balanced, <0.2 |
| 18.5-25 | Binary | 0.0694 | -0.0426 | Balanced, <0.2 |
| ≥25 | Binary | 0.0240 | 0.0213 | Balanced, <0.2 |
| **Resistance category** | | | | |
| RR-TB | Binary | 0.0972 | 0.0000 | Balanced, <0.2 |
| MDR-TB | Binary | 0.0934 | 0.0426 | Balanced, <0.2 |
| pre-XDR-TB | Binary | -0.1907 | -0.0426 | Balanced, <0.2 |
| **Tuberculous pleurisy** | Binary | 0.0833 | -0.0426 | Balanced, <0.2 |
| **Treatment time** **(mo)** | Contin. | -0.2043 | -0.1192 | Balanced, <0.2 |
| **Cavities** | Binary | 0.1288 | -0.0213 | Balanced, <0.2 |
| Cycloserine | Binary | 0.0227 | 0.0638 | Balanced, <0.2 |
| Amikacin/Capreomycin | Binary | -0.0758 | -0.0851 | Balanced, <0.2 |
| Prothionamide | Binary | -0.0795 | -0.0213 | Balanced, <0.2 |
| Balance tally for mean differences | Count |  | | |
| Balanced, <0.2 | 19 |  |  |  |
| Not Balanced, >0.2 | 0 |  |  |  |
| Sample sizes | Control | Treated |  | |
| All | 72 | 88 |  |  |
| Matched | 47 | 47 |  |  |
| Unmatched | 25 | 41 |  |  |

Diff.Un: differences of unadjusted, Diff.Adj: differences of adjusted, M.Threshold: threshold of match, mo: month. BMI: body mass index, RR-TB: rifampicin-resistant tuberculosis, MDR: multidrug-resistant tuberculosis, pre-XDR: pre-extensively drug-resistant tuberculosis.

**Table S3. Adjusted baseline comparison between the 6-month and prolonged groups treated with bedaquiline after PSM**

| Variable | Number (%) | | *P* | SMD |
| --- | --- | --- | --- | --- |
|  | 6mo (n=47) | >6mo (n=47) |  |  |
| **Gender (Male)** | 16 (34.0) | 15 (31.9) | 1.000 | 0.045 |
| **Age** |  | | | |
| <18y | 4 ( 8.5) | 2 ( 4.3) | 0.950 | 0.189 |
| 18-35y | 21 (44.7) | 22 (46.8) |  |  |
| 35-45y | 10 (21.3) | 10 (21.3) |  |  |
| 45-60y | 9 (19.1) | 9 (19.1) |  |  |
| ≥60y | 3 (6.4) | 4 ( 8.5) |  |  |
| **BMI** **(kg/m^2^)** |  | | | |
| <18.5 | 10 (21.3) | 11 (23.4) | 0.854 | 0.137 |
| 18.5-25 | 36 (76.6) | 34 (72.3) |  |  |
| ≥25 | 1 ( 2.1) | 2 ( 4.3) |  |  |
| **Diabetes** | 8 (17.0) | 7 (14.9) | 1.000 | 0.058 |
| **Resistance category** |  | | | |
| RR-TB | 2 ( 4.3) | 2 (4.3) | 0.924 | 0.105 |
| MDR-TB | 9 (19.1) | 11 (23.4) |  |  |
| pre-XDR-TB | 36 (76.6) | 34 (72.3) |  |  |
| **Treatment category** |  | | | |
| New | 16 (34.0) | 13 (27.7) | 0.655 | 0.139 |
| Retreatment | 31(66.0) | 34 (72.3) |  |  |
| **Sputum culture at baseline** (Positive) | 44 (93.6) | 45 (95.7) | 1.000 | 0.095 |
| **Cavities** | 25 (53.2) | 24 (51.1) | 1.000 | 0.043 |
| **Scope of lesions (lobes)** |  | | | |
| 1-2 | 17 (36.2) | 19 (40.4) | 0.753 | 0.156 |
| 3-4 | 12 (25.5) | 9 (19.1) |  |  |
| 5-6 | 18 (38.3) | 19 (40.4) |  |  |
| **Bronchial tuberculosis** | 7 (14.9) | 6 (12.8) | 1.000 | 0.062 |
| **Tuberculous pleurisy** | 3 ( 6.4) | 1 ( 2.1) | 0.617 | 0.212 |
| **Extrapulmonary** | 2 ( 4.3) | 1 ( 2.1) | 1.000 | 0.121 |
| **Clinical symptoms** |  |  |  |  |
| Cough | 32 (68.1) | 25 (53.2) | 0.205 | 0.308 |
| Expectoration | 28 (59.6) | 23 (48.9) | 0.408 | 0.215 |
| Fever | 9 (19.1) | 4 ( 8.5) | 0.232 | 0.312 |
| Hemoptysis | 12 (25.5) | 8 (17.0) | 0.450 | 0.209 |
| Dyspnea | 6 (12.8) | 5 (10.6) | 1.000 | 0.066 |
| **Treatment times (mo), median (IQR)** | 19.0 (16.50, 24.00) | 19.0 (16.00, 20.50) | 0.494 | 0.114 |
| **Drugs** |  |  |  |  |
| Linezolid | 43 (91.5) | 43 (91.5) | 1.000 | <0.001 |
| Fluoroquinolone | 11 (23.4) | 9 (19.1) | 0.801 | 0.104 |
| Clofazimine | 37 (78.7) | 30 (63.8) | 0.171 | 0.334 |
| Cycloserine | 34 (72.3) | 37 (78.7) | 0.631 | 0.149 |
| Pyrazinamide | 22 (46.8) | 26 (55.3) | 0.536 | 0.171 |
| Ethambutol | 5 (10.6) | 5 ( 10.6) | 1.000 | <0.001 |
| Delamanid* | 1 ( 2.1) | 1 ( 2.1) | 1.000 | <0.001 |
| Amikacin/Capreomycin | 21 (44.7) | 17 (36.2) | 0.528 | 0.174 |
| Sodium p-aminosalicylate | 18 (38.3) | 14 (29.8) | 0.514 | 0.180 |
| Prothionamide | 26 (55.3) | 25 (53.2) | 1.000 | 0.043 |

Data are n (%) or median (IQR), SMD: standard mean difference, BMI: body mass index, RR-TB: rifampicin-resistant tuberculosis, MDR: multidrug-resistant tuberculosis, pre-XDR: pre-extensively drug-resistant tuberculosis, mo: month. Percentages calculated based on the number of patients with a result at baseline (baseline refers to 4 weeks before or 2 weeks after treatment initiation).

**Table S4. Baseline comparison between the 6-month group and the 7-9 months group treated with bedaquiline before and after PSM**

| Variable | Number (%)(Unadjusted) | | Unadjusted | | Number (%)(Adjusted) | | Adjusted | |
| --- | --- | --- | --- | --- | --- | --- | --- | --- |
|  | 6mo (n=72) | 7-9mo (n=50) | *P* | SMD | 6mo (n=32) | 7-9mo (n=32) | *P* | SMD |
| **Gender (Male)** | 48 (66.7) | 32 (64.0) | 0.760 | 0.056 | 13 (40.6) | 11(34.4) | 0.796 | 0.129 |
| **Age** |  | | | | | | | |
| <18y | 4 (5.6) | 2 ( 4.0) | 0.894 | 0.204 | 1 (3.1) | 1 (3.1) | 0.971 | 0.199 |
| 18-35y | 33 (45.8) | 25 (50.0) |  |  | 17 (53.1) | 15 (46.9) |  |  |
| 35-45y | 16 (22.2) | 10 (20.0) |  |  | 7 (21.9) | 7 (21.9) |  |  |
| 45-60y | 10 (13.9) | 9 (18.0) |  |  | 3 (9.4) | 5 (15.6) |  |  |
| ≥60y | 9 (12.5) | 4 ( 8.0) |  |  | 4 (12.5) | 4 (12.5) |  |  |
| **BMI (kg/m^2^)** |  | | | |  |  |  |  |
| <18.5 | 19 (26.4) | 7 (11.1) | 0.256 | 0.318 | 5 (15.6) | 5 (15.6) | 1.000 | 0.149 |
| 18.5-25 | 49 (68.1) | 39 (78.0) |  |  | 25 (78.1) | 26 (81.2) |  |  |
| ≥25 | 4 ( 5.6) | 4 ( 8.0) |  |  | 2 (6.2) | 1 (3.1) |  |  |
| **Diabetes** | 9 (12.5) | 8 (16.0) | 0.583 | 0.100 | 5 (15.6) | 6 (18.8) | 1.000 | 0.083 |
| **Resistance category** | | | | | | | | |
| RR-TB | 2 ( 2.8) | 7 (14.0) | **0.006** | 0.595 | 2 (6.2) | 1 (3.1) | 0.833 | 0.191 |
| MDR-TB | 17 (23.6) | 22 (38.0) |  |  | 8 (25.0) | 10 (31.2) |  |  |
| pre-XDR-TB | 53 (73.6) | 32 (48.0) |  |  | 22 (68.8) | 21 (65.6) |  |  |
| **Treatment category** | | | | | | | | |
| New | 25 (34.7) | 12 (24.0) | 0.205 | 0.237 | 12 (37.5) | 8 (25.0) | 0.418 | 0.272 |
| Retreatment | 47 (65.3) | 38 (76.0) |  |  | 20 (62.5) | 24 (75.0) |  |  |
| **Sputum culture at baseline (Positive)** | 68 (94.4) | 44 (88.0) | 0.314 | 0.229 | 30 (93.8) | 31 (96.9) | 1.000 | 0.148 |
| **Cavities** | 30 (41.7) | 24 (48.0) | 0.489 | 0.128 | 16 (50.0) | 15 (46.9) | 1.000 | 0.063 |
| **Scope of lesions (lobes)** | | | | | | | | |
| 1-2 | 29 (40.3) | 30 (60.0) | 0.098 | 0.405 | 14 (43.8) | 18 (56.2) | 0.537 | 0.282 |
| 3-4 | 18 (25.0) | 9 (18.0) |  |  | 8 (25.0) | 5 (15.6) |  |  |
| 5-6 | 25 (34.7) | 11 (22.0) |  |  | 10 (31.2) | 9 (28.1) |  |  |
| **Clinical symptoms** | |  |  |  |  |  |  |  |
| Cough | 46 (63.9) | 29 (58.0) | 0.511 | 0.121 | 19 (59.4) | 20 (62.5) | 1.000 | 0.064 |
| Expectoration | 40 (55.6) | 26 (52.0) | 0.698 | 0.071 | 16 (50.0) | 18 (56.2) | 0.802 | 0.125 |
| Fever | 12 (16.7) | 8 (16.0) | 0.922 | 0.018 | 5 (15.6) | 4 (12.5) | 1.000 | 0.090 |
| Hemoptysis | 17 (23.6) | 9 (18.0) | 0.457 | 0.139 | 7 (21.9) | 7 (21.9) | 1.000 | <0.001 |
| Dyspnea | 6 (8.3) | 8 (16.0) | 0.191 | 0.236 | 1 (3.1) | 4 (12.5) | 0.355 | 0.355 |
| **Treatment times (mo) , median (IQR)** | 18.25 (16.00, 24.00) | 18.0 (12.00, 19.75) | **0.018** | 0.500 | 18.00 (14.00, 21.00) | 18.50 (14.25, 21.00) | 0.995 | 0.006 |
| **Drugs** |  |  |  |  |  |  |  |  |
| Linezolid | 65 (90.3) | 43 (86.0) | 0.466 | 0.133 | 27 (84.4) | 28 (87.5) | 1.000 | 0.090 |
| Fluoroquinolone | 14 (19.4) | 9 (18.0) | 0.841 | 0.037 | 8 (25.0) | 7 (21.9) | 1.000 | 0.074 |
| Clofazimine | 55 (76.4) | 34 (68.0) | 0.305 | 0.188 | 22 (68.8) | 24 (75.0) | 0.781 | 0.139 |
| Cycloserine | 54 (75.0) | 37 (74.0) | 0.901 | 0.023 | 27 (84.4) | 25 (78.1) | 0.749 | 0.161 |
| Pyrazinamide | 36 (50.0) | 27 (54.0) | 0.664 | 0.080 | 17 (53.1) | 16 (50.0) | 1.000 | 0.063 |
| Ethambutol | 6 (8.3) | 5 ( 10.0) | 0.752 | 0.058 | 3 (9.4) | 3 (9.4) | 1.000 | <0.001 |
| Delamanid | 2 ( 2.8) | 3 ( 6.0) | 0.399 | 0.158 | 1 (3.1) | 1 (3.1) | 1.000 | <0.001 |
| Amikacin/Capreomycin | 30 (41.7) | 16 (32.0) | 0.279 | 0.201 | 9 (28.1) | 9 (28.1) | 1.000 | <0.001 |
| Sodium p-aminosalicylate | 27 (37.5) | 11 (22.0) | 0.069 | 0.344 | 11 (34.4) | 8 (25.0) | 0.584 | 0.206 |
| Prothionamide | 45 (62.5) | 29 (58.0) | 0.617 | 0.092 | 24 (75.0) | 19 (59.4) | 0.287 | 0.337 |

Data are n (%) or median (IQR), SMD: standard mean difference, BMI: body mass index, RR-TB: rifampicin-resistant tuberculosis, MDR: multidrug-resistant tuberculosis, pre-XDR: pre-extensively drug-resistant tuberculosis, mo: months, CT: computed tomography. Percentages calculated based on the number of patients with a result at baseline (baseline refers to 4 weeks before or 2 weeks after treatment initiation).

**Table S5. Correlation analysis of factors affecting treatment outcomes (6-month and 7-9 months groups)**

| Dependent: Outcome | | Success  (n=97) | Failure  (n=25) | OR (univariable)  (95% CI，*P*） |
| --- | --- | --- | --- | --- |
| **Gender** | | | | |
| Male | | 65 (67.0) | 15 (60.0) |  |
| Female | | 32 (33.0) | 10 (40.0) | 1.35 (0.55-3.35, *P*=0.511) |
| **Age** | | | | |
| <18y | | 5 (5.2) | 1 (4.0) |  |
| 18-35y | | 47 (48.5) | 11 (44.0) | 1.17 (0.12-11.05, *P*=0.891) |
| 35-45y | | 19 (19.6) | 7 (28.0) | 1.84 (0.18-18.66, *P*=0.605) |
| 45-60y | | 15 (15.5) | 4 (16.0) | 1.33 (0.12-14.90, *P*=0.815) |
| ≥60y | | 11 (11.3) | 2 (8.0) | 0.91 (0.07-12.52, *P*=0.943) |
| **BMI (kg/m^2^)** |  |  |  |  |
| <18.5 | | 18 (18.6) | 8 (32.0) |  |
| 18.5-25 | | 71 (73.2) | 17 (68.0) | 0.54 (0.20-1.45, *P*=0.219) |
| ≥25 | | 8 (8.2) | 0 (0) | 0.00 (0.00-Inf, *P*=0.990) |
| **Treatment times (mo), Mean ± SD** | | 17.5 ± 4.6 | 21.7 ± 7.2 | **1.15 (1.05-1.25, *P*=0.002)** |
| **Resistance category** |  |  |  |  |
| RR-TB | | 8 (8.2) | 1 (4.0) |  |
| MDR-TB | | 31 (32.0) | 5 (20.0) | 1.29 (0.13-12.66, *P*=0.827) |
| pre-XDR-TB | | 58 (59.8) | 19 (76.0) | 2.62 (0.31-22.33, *P*=0.378) |
| **Bronchial tuberculosis** | | 15 (15.5) | 5 (20.0) | 1.37 (0.44-4.21, *P*=0.586) |
| **Tuberculous pleurisy** | | 4 (4.1) | 4 (16.0) | **4.43 (1.02-19.16, *P*=0.046)** |
| **Extrapulmonary** | | 1 (1.0) | 1 (4.0) | 4.00 (0.24-66.28, *P*=0.333) |
| **Treatment category** | | | | |
| New | | 28 (28.9) | 9 (36.0) |  |
| Retreatment | | 69 (71.1) | 16 (64.0) | 0.72 (0.29-1.82, *P*=0.490) |
| **Diabetes** | | 12 (12.4) | 5 (20.0) | 1.77 (0.56-5.60, *P*=0.331) |
| **Culture at baseline (Positive)** | | 89 (91.8) | 23 (92.0) | 1.03 (0.21-5.20, *P*=0.968) |
| **Clinical symptoms** | |  |  |  |
| Cough | | 59 (60.8) | 16 (64.0) | 1.15 (0.46-2.85, *P*=0.771) |
| Expectoration | | 53 (54.6) | 13 (52.0) | 0.90 (0.37-2.17, *P*=0.813) |
| Fever | | 17 (17.5) | 3 (12.0) | 0.64 (0.17-2.39, *P*=0.508) |
| Hemoptysis | | 21 (21.6) | 5 (20.0) | 0.90 (0.30-2.70, *P*=0.858) |
| Dyspnea | | 11 (11.3) | 3 (12.0) | 1.07 (0.27-4.15, *P*=0.926) |
| **Cavities** | | 37 (38.1) | 17 (68.0) | **3.45 (1.35-8.78, *P*=0.009)** |
| **Scope of lesions (lobes)** |  |  |  |  |
| 1-2 | | 50 (51.5) | 9 (36.0) |  |
| 3-4 | | 19 (19.6) | 8 (32.0) | 2.34 (0.79-6.95, *P*=0.126) |
| 5-6 | | 28 (28.9) | 8 (32.0) | 1.59 (0.55-4.58, *P*=0.392) |
| **Drugs** | |  |  |  |
| Linezolid | | 85 (87.6) | 23 (92.0) | 1.62 (0.34-7.77, *P*=0.544) |
| Fluoroquinolone | | 18 (18.6) | 5 (20.0) | 1.10 (0.36-3.31, *P*=0.869) |
| Clofazimine | | 68 (70.1) | 21 (84.0) | 2.24 (0.71-7.10, *P*=0.171) |
| Cycloserine | | 77 (79.4) | 14 (56.0) | **0.33 (0.13-0.84, *P*=0.020)** |
| Pyrazinamide | | 51 (52.6) | 12 (48.0) | 0.83 (0.35-2.01, *P*=0.683) |
| Ethambutol | | 7 (7.2) | 4 (16.0) | 2.45 (0.66-9.14, *P*=0.183) |
| Delamanid | | 5 (5.2) | 0 (0.0) | 0.00 (0.00-Inf, *P*=0.989) |
| Amikacin/Capreomycin | | 32 (33.0) | 14 (56.0) | **2.59 (1.06-6.33, *P*=0.038)** |
| Sodium p-aminosalicylate | | 31 (32.0) | 7 (28.0) | 0.83 (0.31-2.19, *P*=0.703) |
| Prothionamide | | 62 (63.9) | 12 (48.0) | 0.52 (0.21-1.27, *P*=0.150) |

Data are n (%) or Mean **±** SD, SD: standard deviation, OR: odds ratio, mo: month. BMI: body mass index, RR-TB: rifampicin-resistant tuberculosis, MDR: multidrug-resistant tuberculosis, pre-XDR: pre-extensively drug-resistant tuberculosis, mo: months.

# Table S6. Balance test of PSM (6-month and 7-9 months groups)

|  | Type | Diff.Un | | Diff.Adj | M.Threshold |
| --- | --- | --- | --- | --- | --- |
| distance | Distance | 0.9155 | | 0.0114 |  |
| **Gender** | Binary | 0.0267 | | -0.0625 | Balanced, <0.2 |
| **Age** |  | | | | |
| <18y | Binary | -0.0156 | | 0.0000 | Balanced, <0.2 |
| 18-35y | Binary | 0.0417 | | -0.0625 | Balanced, <0.2 |
| 35-45 | Binary | -0.0222 | | 0.0000 | Balanced, <0.2 |
| 45-60 | Binary | 0.0411 | | 0.0625 | Balanced, <0.2 |
| ≥60y | Binary | -0.0450 | | 0.0000 | Balanced, <0.2 |
| **BMI (kg/m^2^)** |  | | | | |
| <18.5 | Binary | -0.1239 | | 0.0000 | Balanced, <0.2 |
| 18.5-25 | Binary | 0.0994 | | 0.0312 | Balanced, <0.2 |
| ≥25 | Binary | 0.0244 | | -0.0312 | Balanced, <0.2 |
| **Resistance category** |  | | | | |
| RR-TB | Binary | 0.1122 | | -0.0312 | Balanced, <0.2 |
| MDR-TB | Binary | 0.1439 | | 0.0625 | Balanced, <0.2 |
| pre-XDR-TB | Binary | -0.2561 | | -0.0312 | Balanced, <0.2 |
| **Treatment times (mo)** | Contin. | -0.5432 | | -0.0065 | Balanced, <0.2 |
| **Cavities** | Binary | 0.0633 | | -0.0312 | Balanced, <0.2 |
| **Tuberculous pleurisy** | Binary | 0.0583 | | 0.0312 | Balanced, <0.2 |
| Cycloserine | Binary | -0.0100 | | -0.0625 | Balanced, <0.2 |
| Amikacin/Capreomycin | Binary | -0.0967 | | 0.0000 | Balanced, <0.2 |
| Balance tally for mean differences | Count |  | | | |
| Balanced, <0.2 | 18 |  |  |  |  |
| Not Balanced, >0.2 | 0 |  |  |  |  |
| Sample sizes | Control | Treated |  | | |
| All | 72 | 50 |  |  |  |
| Matched | 32 | 32 |  |  |  |
| Unmatched | 40 | 18 |  | |  |

Diff.Un: differences of unadjusted, Diff.Adj: differences of adjusted, M.Threshold: threshold of match, mo: month. BMI: body mass index, RR-TB: rifampicin-resistant tuberculosis, MDR: multidrug-resistant tuberculosis, pre-XDR: pre-extensively drug-resistant tuberculosis.

**Table S7. Baseline comparison between the 6-month group and 10-12 months group treated with bedaquiline before and after PSM**

| Variable | Number (%) (Unadjusted) | | Unadjusted | | Number (%) (Adjusted) | | Adjusted | |
| --- | --- | --- | --- | --- | --- | --- | --- | --- |
|  | 6mo (n=72) | 10-12mo (n=21) | *P* | SMD | 6mo (n=10) | 10-12mo (n=10) | *P* | SMD |
| **Gender (Male)** | 48 (66.7) | 15 ( 71.4) | 0.681 | 0.103 | 2 (20.0) | 3 (30.0) | 1.000 | 0.232 |
| **Age** | | | | | | | | |
| <18y | 4 ( 5.6) | 1(4.8) | 0.325 | 0.632 | 0 (0.0) | 1 (10.0) | 1.000 | 0.535 |
| 18-35y | 33 (45.8) | 12 (57.1) |  |  | 5 (50.0) | 5 (50.0) |  |  |
| 35-45y | 16 (22.2) | 3 (14.3) |  |  | 3 (30.0) | 3 (30.0) |  |  |
| 45-60y | 10 (13.9) | 5 (23.8) |  |  | 2 (20.0) | 1 (10.0) |  |  |
| ≥60y | 9 (12.5) | 0 (0.0) |  |  | 0 (0.0) | 0 (0.0) |  |  |
| **BMI** **(kg/m^2^)** | | | | | | | | |
| <18.5 | 19 (26.4) | 4 (19.0) | 0.699 | 0.216 | 1 (10.0) | 1 (10.0) | 1.000 | 0.473 |
| 18.5-25 | 49 (68.1) | 15 (71.4) |  |  | 9 (90.0) | 8 (80.0) |  |  |
| ≥25 | 4 ( 5.6) | 2 (9.5) |  |  | 0 (0.0) | 1 (10.0) |  |  |
| **Diabetes** | 9 (12.5) | 2 (9.5) | 1.000 | 0.095 | 1 (10.0) | 1 (8.3) | 1.000 | 0.471 |
| **Resistance category** | | | | | | | | |
| RR-TB | 2 ( 2.8) | 3 (14.3) | 0.056 | 0.527 | 1 (10.0) | 1 (10.0) | 1.000 | <0.001 |
| MDR-TB | 17 (23.6) | 7 (33.3) |  |  | 3 (30.0) | 3 (30.0) |  |  |
| pre-XDR-TB | 53 (73.6) | 11 (52.4) |  |  | 6 (60.0) | 6 (60.0) |  |  |
| **Bronchial tuberculosis** | 12 (16.7) | 2 (9.5) | 0.729 | 0.213 | 0 (0.0) | 2 (20.0) | 0.474 | 0.707 |
| **Tuberculous pleurisy** | 3 ( 4.2) | 4 (19.0) | **0.044** | 0.478 | 0 (0.0) | 0 (0.0) | 1.000 | <0.001 |
| **Extrapulmonary*** | 2 ( 2.8) | 2 (9.5) | 0.219 | 0.284 | 0 (0.0) | 0 (0.0) | 1.000 | <0.001 |
| **Treatment category** | | | | | | | | |
| New | 25 (34.7) | 7 (33.3) | 0.906 | 0.029 | 5 (50.0) | 2 (20.0) | 0.350 | 0.663 |
| Retreatment | 47 (65.3) | 14 (66.7) |  |  | 5 (50.0) | 8 (80.0) |  |  |
| **Culture at baseline** (Positive) | 68 (94.4) | 18 (85.7) | 0.188 | 0295 | 9 (90.0) | 9 (90.0) | 1.000 | <0.001 |
| **Cavities** | 30 (41.7) | 13 ( 61.9) | 0.102 | 0.414 | 4 (40.0) | 5 (50.0) | 1.000 | 0.202 |
| **Scope of lesions (lobes)** | | | | | | | | |
| 1-2 | 29 (40.3) | 7 (33.3) | 0.631 | 0.266 | 6 (60.0) | 4 (40.0) | 0.714 | 0.428 |
| 3-4 | 18 (25.0) | 4 (19.0) |  |  | 1 (10.0) | 2 (20.0) |  |  |
| 5-6 | 25 (34.7) | 10 (47.6) |  |  | 3 (30.0) | 4 (40.0) |  |  |
| **Clinical symptoms** |  |  |  |  |  |  |  |  |
| Cough | 46 (63.9) | 10 (47.6) | 0.180 | 0.332 | 5 (50.0) | 4 (40.0) | 1.000 | 0.202 |
| Expectoration | 40 (55.6) | 7 (33.3) | 0.073 | 0.459 | 4 (40.0) | 2 (20.0) | 0.628 | 0.447 |
| Fever | 12 (16.7) | 4 (19.0) | 0.752 | 0.062 | 2 (20.0) | 0 (0.0) | 0.474 | 0.707 |
| Hemoptysis | 17 (23.6) | 2 (9.5) | 0.224 | 0.386 | 1 (10.0) | 2 (20.0) | 1.000 | 0.283 |
| Dyspnea | 6 (8.3) | 2 (9.5) | 1.000 | 0.042 | 0 (0.0) | 1 (10.0) | 1.000 | 0.471 |
| **Treatment times (mo) , median(IQR)** | 18.25 (16.00, 24.00) | 18.0 (16.00, 20.00) | 0.486 | 0.085 | 18.0 (15.50, 20.25) | 18.5 (16.50, 21.50) | 0.646 | 0.072 |
| **Drugs** |  |  |  |  |  |  |  |  |
| Linezolid | 65 (90.3) | 19 (90.5) | 0.978 | 0.007 | 10 (100.0) | 9 (90.0) | 1.000 | 0.471 |
| Fluoroquinolone | 14 (19.4) | 8 (38.1) | 0.077 | 0.421 | 0 (0.0) | 3 (30.0) | 0.211 | 0.926 |
| Clofazimine | 55 (76.4) | 12 (57.1) | 0.084 | 0.417 | 7 (70.0) | 5 (50.0) | 0.650 | 0.417 |
| Cycloserine | 54 (75.0) | 17 (81.0) | 0.772 | 0.144 | 8 (80.0) | 9 (90.0) | 1.000 | 0.283 |
| Pyrazinamide | 36 (50.0) | 13 ( 61.9) | 0.336 | 0.242 | 6 (60.0) | 6 (60.0) | 1.000 | <0.001 |
| Ethambutol | 6 (8.3) | 0 (0.0) | 0.331 | 0.426 | 0 (0.0) | 0 (0.0) | 1.000 | <0.001 |
| Delamanid | 2 ( 2.8) | 0 (0.0) | 1.000 | 0.239 | 0 (0.0) | 0 (0.0) | 1.000 | <0.001 |
| Amikacin/Capreomycin | 30 (41.7) | 3 (14.3) | **0.021** | 0.640 | 2 (20.0) | 2 (20.0) | 1.000 | <0.001 |
| Sodium p-aminosalicylate | 27 (37.5) | 7 (33.3) | 0.727 | 0.087 | 3 (30.0) | 4 (40.0) | 1.000 | 0.211 |
| Prothionamide | 45 (62.5) | 13 (61.9) | 0.960 | 0.012 | 8 (80.0) | 6 (60.0) | 0.628 | 0.447 |

Data are n (%) or median (IQR), SMD: standard mean difference, BMI: body mass index, RR-TB: rifampicin-resistant tuberculosis, MDR: multidrug-resistant tuberculosis, pre-XDR: pre-extensively drug-resistant tuberculosis, mo: month.

**Table S8. Correlation analysis of factors affecting treatment outcomes (6-month and 10-12 months groups)**

| Dependent: Outcome | Success  (n=74) | Failure  (n=19) | OR (univariable)  (95% CI，*P*） |
| --- | --- | --- | --- |
| **Gender** | | | |
| Male | 52 (70.3) | 11 (57.9) |  |
| Female | 22 (29.7) | 8 (42.1) | 1.72 (0.61-4.85, *P*=0.306) |
| **Age** | | | |
| <18y | 4 (5.4) | 1 (5.3) |  |
| 18-35y | 36 (48.6) | 9 (47.4) | 1.00 (0.10-10.07, *P*=1.000) |
| 35-45y | 12 (16.2) | 7 (36.8) | 2.33 (0.22-25.24, *P*=0.486) |
| 45-60y | 13 (17.6) | 2 (10.5) | 0.62 (0.04-8.70, *P*=0.719) |
| ≥60y | 9 (12.2) | 0 (0.0) | 0.00 (0.00-Inf, *P*=0.990) |
| **BMI (kg/m^2^)** | | | |
| <18.5 | 18 (24.3) | 5 (26.3) |  |
| 18.5-25 | 50 (67.6) | 14 (73.7) | 1.01 (0.32-3.20, *P*=0.989) |
| ≥25 | 6 (8.1) | 0 (0.0) | 0.00 (0.00-Inf, *P*=0.992) |
| **Treatment times (mo), Mean ± SD** | 18.5 ± 4.9 | 22.6 ± 7.0 | **1.13 (1.03-1.24, *P*=0.009)** |
| **Resistance category** | | | |
| RR-TB | 4 (5.4) | 1 (5.3) |  |
| MDR-TB | 21 (28.4) | 3 (15.8) | 0.57 (0.05-6.98, *P*=0.661) |
| pre-XDR-TB | 49 (66.2) | 15 (78.9) | 1.22 (0.13-11.81, *P*=0.861) |
| **Bronchial tuberculosis** | 10 (13.5) | 4 (21.1) | 1.71 (0.47-6.19, *P*=0.416) |
| **Tuberculous pleurisy** | 5 (6.8) | 2 (10.5) | 1.62 (0.29-9.10, *P*=0.582) |
| **Extrapulmonary** | 3 (4.1) | 1 (5.3) | 1.31 (0.13-13.40, *P*=0.817) |
| **Treatment category** | | | |
| New | 24 (32.4) | 8 (42.1) |  |
| Retreatment | 50 (67.6) | 11 (57.9) | 0.66 (0.24-1.85, *P*=0.430) |
| **Diabetes** | 9 (12.2) | 2 (10.5) | 0.85 (0.17-4.30, *P*=0.844) |
| **Culture at baseline (**Positive) | 69 (93.2) | 17 (89.5) | 0.62 (0.11-3.45, *P*=0.582) |
| **Clinical symptoms** |  |  |  |
| Cough | 44 (59.5) | 12 (63.2) | 1.17 (0.41-3.31, *P*=0.769) |
| Expectoration | 37 (50.0) | 10 (52.6) | 1.11 (0.40-3.05, *P*=0.838) |
| Fever | 13 (17.6) | 3 (15.8) | 0.88 (0.22-3.47, *P*=0.855) |
| Hemoptysis | 17 (23.0) | 2 (10.5) | 0.39 (0.08-1.88, *P*=0.243) |
| Dyspnea | 6 (8.1) | 2 (10.5) | 1.33 (0.25-7.20, *P*=0.738) |
| **Cavities** | 30 (40.5) | 13 (68.4) | **3.18 (1.09-9.29, *P*=0.035)** |
| **Scope of lesions (lobes)** | | | |
| 1-2 | 30 (40.5) | 6 (31.6) |  |
| 3-4 | 15 (20.3) | 7 (36.8) | 2.33 (0.67-8.18, *P*=0.185) |
| 5-6 | 29 (39.2) | 6 (31.6) | 1.03 (0.30-3.58, *P*=0.957) |
| **Drug** |  |  |  |
| Linezolid | 66 (89.2) | 18 (94.7) | 2.18 (0.26-18.60, *P*=0.476) |
| Fluoroquinolone | 17 (23) | 5 (26.3) | 1.20 (0.38-3.80, *P*=0.760) |
| Clofazimine | 53 (71.6) | 14 (73.7) | 1.11 (0.36-3.47, *P*=0.858) |
| Cycloserine | 59 (79.7) | 12 (63.2) | 0.44 (0.15-1.30, *P*=0.136) |
| Pyrazinamide | 40 (54.1) | 9 (47.4) | 0.76 (0.28-2.10, *P*=0.603) |
| Ethambutol | 4 (5.4) | 2 (10.5) | 2.06 (0.35-12.19, *P*=0.426) |
| Delamanid | 2 (2.7) | 0 (0.0) | 0.00 (0.00-Inf, *P*=0.993) |
| Amikacin/Capreomycin | 21 (28.4) | 12 (63.2) | **4.33 (1.50-12.49, *P*=0.007)** |
| Sodium p-aminosalicylate | 28 (37.8) | 6 (31.6) | 0.76 (0.26-2.22, *P*=0.614) |
| Prothionamide | 49 (66.2) | 9 (47.4) | 0.46 (0.17-1.28, *P*=0.135) |

Data are n (%) or Mean **±** SD, SD: standard deviation, OR: odds ratio, mo: month. BMI: body mass index, RR-TB: rifampicin-resistant tuberculosis, MDR: multidrug-resistant tuberculosis, pre-XDR: pre-extensively drug-resistant tuberculosis.

**Table S9. Balance test of PSM (6-month and 10-12 months groups)**

|  | Type | Diff.Un | | Diff.Adj | M.Threshold |
| --- | --- | --- | --- | --- | --- |
| distance | Distance | 1.3394 | | 0.0003 |  |
| **Gender** | Binary | -0.0476 | | 0.1429 | Balanced, <0.2 |
| **Age** |  |  | |  |  |
| <18y | Binary | -0.0079 | | 0.0714 | Balanced, <0.2 |
| 18-35y | Binary | 0.1131 | | 0.0714 | Balanced, <0.2 |
| 35-45 | Binary | -0.0794 | | 0.0000 | Balanced, <0.2 |
| 45-60 | Binary | 0.0992 | | -0.1429 | Balanced, <0.2 |
| ≥60y | Binary | -0.1250 | | 0.0000 | Balanced, <0.2 |
| **BMI (**kg/m^2^) |  |  | |  |  |
| <18.5 | Binary | -0.0734 | | -0.0714 | Balanced, <0.2 |
| 18.5-25 | Binary | 0.0337 | | 0.0714 | Balanced, <0.2 |
| ≥25 | Binary | 0.0397 | | 0.0000 | Balanced, <0.2 |
| **Resistance category** |  |  | |  |  |
| RR-TB | Binary | 0.1151 | | -0.0714 | Balanced, <0.2 |
| MDR-TB | Binary | -0.0972 | | 0.0714 | Balanced, <0.2 |
| pre-XDR-TB | Binary | -0.2123 | | 0.0000 | Balanced, <0.2 |
| **Tuberculous pleurisy** | Binary | 0.1488 | | 0.0714 | Balanced, <0.2 |
| Hemoptysis | Binary | -0.1409 | | 0.1000 | Balanced, <0.2 |
| **Treatment times (mo)** | Contin. | -0.0870 | | 0.1469 | Balanced, <0.2 |
| **Cavities** | Binary | 0.2024 | | -0.0714 | Balanced, <0.2 |
| Amikacin/Capreomycin | Binary | -0.2738 | | -0.0714 | Balanced, <0.2 |
| Balance tally for mean differences | Count |  | | | |
| Balanced, <0.2 | 18 |  |  |  |  |
| Not Balanced, >0.2 | 0 |  |  |  |  |
| Sample sizes | Control | Treated |  | | |
| All | 72 | 21 |  |  |  |
| Matched | 10 | 10 |  |  |  |
| Unmatched | 62 | 11 |  |  |  |

Diff.Un: differences of unadjusted, Diff.Adj: differences of adjusted, M.Threshold: threshold of match, mo: month. BMI: body mass index, RR-TB: rifampicin-resistant tuberculosis, MDR: multidrug-resistant tuberculosis, pre-XDR: pre-extensively drug-resistant tuberculosis.

**Table S10. Baseline comparison between the 6-month group and >12 months group treated with bedaquiline before and after PSM**

| Variable | | Number (%) (Unadjusted) | | Unadjusted | | Number (%) (Adjusted) | | Adjusted | |
| --- | --- | --- | --- | --- | --- | --- | --- | --- | --- |
|  |  | 6mo (n=72) | >12mo (n=17) | *P* | SMD | 6mo (n=11) | >12mo (n=11) | *P* | SMD |
| **Gender (Male)** | | 48 (66.7) | 12 (70.6) | 0.756 | 0.085 | 6 (54.5) | 4 (36.4) | 0.670 | 0.371 |
| **Age** | | | | | | | | | |
| <18y | | 4 (5.6) | 0 (0.0) | 0.475 | 0.575 | 0 (0.0) | 0 (0.0) | 0.850 | 0.407 |
| 18-35y | | 33 (45.8) | 10 (58.8) |  |  | 6 (54.5) | 4 (36.4) |  |  |
| 35-45y | | 16 (22.2) | 2 (11.8) |  |  | 1 (9.1) | 2 (18.2) |  |  |
| 45-60y | | 10 (13.9) | 4 (23.5) |  |  | 3 (27.3) | 4 (36.4) |  |  |
| ≥60y | | 9 (12.5) | 1 (5.9) |  |  | 1 (9.1) | 1 (9.1) |  |  |
| **BMI** (kg/m^2^) | | | | | | | | | |
| <18.5 | | 19 (26.4) | 4 (23.5) | 0.971 | 0.066 | 4 (36.4) | 3 (27.3) | 0.659 | 0.527 |
| 18.5-25 | | 49 (68.1) | 12 (70.6) |  |  | 6(54.5) | 8 (72.7) |  |  |
| ≥25 | | 4 (5.6) | 1 (5.9) |  |  | 1 (9.1) | 0 (0.0) |  |  |
| **Diabetes** | | 9 (12.5) | 1 (5.9) | 0.437 | 0.231 | 0 (0.0) | 1 (9.1) | 1.000 | 0.447 |
| **Resistance category** | | | | | | | | | |
| RR-TB | | 2 (2.8) | 1 (5.9) | 0.731 | 0.203 | 1 (9.1) | 0 (0.0) | 1.000 | 0.508 |
| MDR-TB | | 17 (23.6) | 3 (17.6) |  |  | 1 (9.1) | 2 (18.2) |  |  |
| pre-XDR-TB | | 53 (73.6) | 13 (76.5) |  |  | 9 (81.8) | 9 (81.8) |  |  |
| **Bronchial tuberculosis** | | 12 (16.7) | 2 (11.8) | 0.618 | 0.141 | 1 (9.1) | 1 (9.1) | 1.000 | <0.001 |
| **Tuberculous pleurisy** | | 3 (4.2) | 4 (19.0) | 0.221 | 0.283 | 0 (0.0) | 0 (0.0) | 1.000 | <0.001 |
| **Extrapulmonary** | | 2 (2.8) | 1 (5.9) | 0.524 | 0.153 | 0 (0.0) | 0 (0.0) | 1.000 | <0.001 |
| **Treatment category** | | | | | | | | | |
| New | | 25 (34.7) | 4 (23.5) | 0.376 | 0.248 | 4 (34.4) | 3 (27.3) | 1.000 | 0.196 |
| Retreatment | | 47 (65.3) | 13 (76.5) |  |  | 7 (63.6) | 8 (72.7) |  |  |
| **Culture at baseline (Positive)** | | 68 (94.4) | 16 (94.1) | 0.958 | 0.014 | 11 (100.0) | 11 (100.0) | 1.000 | <0.001 |
| **Cavities** | | 30 (41.7) | 11 (64.7) | 0.087 | 0.475 | 7 (63.6) | 7 (63.6) | 1.000 | <0.001 |
| **Scope of lesions (lobes)** | | | | | | | | | |
| 1-2 | | 29 (40.3) | 2 (11.8) | 0.069 | 0.707 | 5 ( 45.5) | 1 (9.1) | 0.225 | 0.915 |
| 3-4 | | 18 (25.0) | 5 (29.4) |  |  | 3 ( 27.3) | 4 (36.4) |  |  |
| 5-6 | | 25 (34.7) | 10 (58.8) |  |  | 3 ( 27.3) | 6 (54.5) |  |  |
| **Clinical symptoms** | | |  |  |  |  |  |  |  |
| Cough | | 46 (63.9) | 9 (52.9) | 0.403 | 0.224 | 8 (72.7) | 6 (54.5) | 0.659 | 0.385 |
| Expectoration | | 40 (55.6) | 8 (47.1) | 0.527 | 0.171 | 5 (45.5) | 5 (45.5) | 1.000 | <0.001 |
| Fever | | 12 (16.7) | 3 (17.6) | 1.000 | 0.026 | 2 (18.2) | 1 (9.1) | 1.000 | 0.267 |
| Hemoptysis | | 17 (23.6) | 3 (17.6) | 0.753 | 0.148 | 2 (18.2) | 1 (9.1) | 1.000 | 0.267 |
| Dyspnea | | 6 ( 8.3) | 1 (5.9) | 1.000 | 0.095 | 0 (0.0) | 0 (0.0) | 1.000 | <0.001 |
| **Treatment times (mo), median(IQR)** | | 18.25 (16.00, 24.00) | 20.0 (17.00, 22.00) | 0.391 | 0.026 | 23.00 (17.00, 26.25) | 20 (18.00, 21.50) | 0.576 | 0.030 |
| **Drugs** | |  |  |  |  |  |  |  |  |
| Linezolid | 65 (90.3) | | 15 (88.2) | 0.802 | 0.066 | 10 (90.9) | 10 (90.9) | 1.000 | <0.001 |
| Fluoroquinolone | 14 (19.4) | | 4 (23.5) | 0.706 | 0.100 | 1 (9.1) | 3 (27.3) | 0.586 | 0.485 |
| Clofazimine | 55 (76.4) | | 12 (70.6) | 0.618 | 0.132 | 8 (72.7) | 7 (63.6) | 1.000 | 0.196 |
| Cycloserine | 54 (75.0) | | 14 (82.4) | 0.521 | 0.180 | 7 (63.6) | 8 (72.7) | 1.000 | 0.196 |
| Pyrazinamide | 36 (50.0) | | 6 (35.3) | 0.275 | 0.301 | 7 (63.6) | 5 (45.5) | 0.670 | 0.371 |
| Ethambutol | 6 (8.3) | | 1 (5.9) | 0.736 | 0.095 | 0 (0.0) | 1 (9.1) | 1.000 | 0.447 |
| Delamanid | 2 (2.8) | | 0 (0.0) | 0.487 | 0.239 | 0 (0.0) | 0 (0.0) | 1.000 | <0.001 |
| Amikacin/Capreomycin | 30 (41.7) | | 11 (64.7) | 0.087 | 0.475 | 7 (63.6) | 7 (63.6) | 1.000 | <0.001 |
| Sodium p-aminosalicylate | 27 (37.5) | | 8 (47.1) | 0.468 | 0.194 | 4 (36.4) | 4 (36.4) | 1.000 | <0.001 |
| Prothionamide | 45 (62.5) | | 6 (35.3) | **0.041** | 0.566 | 4 (36.4) | 4 (36.4) | 1.000 | <0.001 |

Data are n (%) or median (IQR), SMD: standard mean difference, BMI: body mass index, RR-TB: rifampicin-resistant tuberculosis, MDR: multidrug-resistant tuberculosis, pre-XDR: pre-extensively drug-resistant tuberculosis, mo: month, CT: computed tomography. Percentages calculated based on the number of patients with a result at baseline (baseline refers to 4 weeks before or 2 weeks after treatment initiation).

**Table S11. Correlation analysis of factors affecting treatment outcomes (6-month and >12 months groups)**

| Dependent: Outcome |  | Success  (n=66) | Failure  (n=23) | OR (univariable)  (95% CI，*P*） |
| --- | --- | --- | --- | --- |
| **Gender** | | | | |
| Male | | 43 (65.2) | 17 (73.9) |  |
| Female | | 23 (34.8) | 6 (26.1) | 0.66 (0.23-1.90, *P*=0.442) |
| **Age** | | | | |
| <18y | | 3 (4.5) | 1 (4.3) |  |
| 18-35y | | 32 (48.5) | 11 (47.8) | 1.03 (0.10-10.97, *P*=0.980) |
| 35-45y | | 12 (18.2) | 6 (26.1) | 1.50 (0.13-17.67, *P*=0.747) |
| 45-60y | | 10 (15.2) | 4 (17.4) | 1.20 (0.09-15.26, *P*=0.888) |
| ≥60y | | 9 (13.6) | 1 (4.3) | 0.33 (0.02-7.14, *P*=0.482) |
| **BMI (kg/m^2^)** | | | | |
| <18.5 | | 16 (24.2) | 7 (30.4) |  |
| 18.5-25 | | 46 (69.7) | 15 (65.2) | 0.75 (0.26-2.16, *P*=0.588) |
| ≥25 | | 4 (6.1) | 1 (4.3) | 0.57 (0.05-6.08, *P*=0.643) |
| **Treatment times (mo), Mean ± SD** | | 18.6 ± 4.9 | 23.5 ± 7.9 | **1.14 (1.04-1.25, *P*=0.003)** |
| **Resistance category** | | | | |
| RR-TB | | 3 (4.5) | 0 (0.0) |  |
| MDR-TB | | 17 (25.8) | 3 (13.0) | 2762004.86 (0.00-Inf, *P*=0.992) |
| pre-XDR-TB | | 46 (69.7) | 20 (87.0) | 6804939.50 (0.00-Inf, *P*=0.991) |
| **Bronchial tuberculosis** | | 9 (13.6) | 5 (21.7) | 1.76 (0.52-5.93, *P*=0.362) |
| **Tuberculous pleurisy** | | 2 (3.0) | 3 (13.0) | 4.80 (0.75-30.78, *P*=0.098) |
| **Extrapulmonary** | | 2 (3.0) | 1 (4.3) | 1.45 (0.13-16.84, *P*=0.764) |
| **Treatment category** | | | | |
| New | | 21 (31.8) | 8 (34.8) |  |
| Retreatment | | 45 (68.2) | 15 (65.2) | 0.88 (0.32-2.38, *P*=0.794) |
| **Diabetes** |  | 7 (10.6) | 3 (13.0) | 1.26 (0.30-5.36, *P*=0.750) |
| **Culture at baseline (Positive)** | | 62 (93.9) | 22 (95.7) | 1.42 (0.15-13.39, *P*=0.760) |
| Cough | | 40 (60.6) | 15 (65.2) | 1.22 (0.45-3.28, *P*=0.695) |
| Expectoration | | 35 (53.0) | 13 (56.5) | 1.15 (0.44-2.99, *P*=0.772) |
| Fever | | 10 (15.2) | 5 (21.7) | 1.56 (0.47-5.15, *P*=0.470) |
| Hemoptysis | | 16 (24.2) | 4 (17.4) | 0.66 (0.19-2.22, *P*=0.500) |
| Dyspnea | | 4 (6.1) | 3 (13.0) | 2.32 (0.48-11.28, *P*=0.295) |
| Cavities | | 25 (37.9) | 16 (69.6) | **3.75 (1.35-10.37, *P*=0.011)** |
| **Scope of lesions (lobes)** | | | | |
| 1-2 | | 26 (39.4) | 5 (21.7) |  |
| 3-4 | | 16 (24.2) | 7 (30.4) | 2.28 (0.62-8.40, *P*=0.217) |
| 5-6 | | 24 (36.4) | 11 (47.8) | 2.38 (0.72-7.86, *P*=0.154) |
| **Treatment times (mo), Mean ± SD** | | 18.6 ± 4.9 | 23.5 ± 7.9 | **1.14 (1.04-1.25, *P*=0.003)** |
| **Drugs** | |  |  |  |
| Linezolid | | 59 (89.4) | 21 (91.3) | 1.25 (0.24-6.48, *P*=0.794) |
| Fluoroquinolone | | 12 (18.2) | 6 (26.1) | 1.59 (0.52-4.87, *P*=0.419) |
| Clofazimine | | 47 (71.2) | 20 (87.0) | 2.70 (0.72-10.14, *P*=0.143) |
| Cycloserine | | 54 (81.8) | 14 (60.9) | **0.35 (0.12-0.98, *P*=0.046)** |
| Pyrazinamide | | 33 (50.0) | 9 (39.1) | 0.64 (0.24-1.69, *P*=0.370) |
| Ethambutol | | 4 (6.1) | 3 (13) | 2.32 (0.48-11.28, *P*=0.295) |
| Delamanid | | 2 (3.0) | 0 (0.0) | 0.00 (0.00-Inf, *P*=0.993) |
| Amikacin/Capreomycin | | 25 (37.9) | 16 (69.6) | **3.75 (1.35-10.37, *P*=0.011)** |
| Sodium p-aminosalicylate | | 25 (37.9) | 10 (43.5) | 1.26 (0.48-3.30, *P*=0.636) |
| Prothionamide | | 39 (59.1) | 12 (52.2) | 0.76 (0.29-1.96, *P*=0.564) |
| Data are n (%) or Mean **±** SD, SD: standard deviation, OR: odds ratio, mo: month. BMI: body mass index, RR-TB: rifampicin-resistant tuberculosis, MDR: multidrug-resistant tuberculosis, pre-XDR: pre-extensively drug-resistant tuberculosis. | | | | |

**Table S12. Balance test of PSM (6-month and >12 months groups)**

|  | Type | Diff.Un | | | Diff.Adj | M.Threshold |
| --- | --- | --- | --- | --- | --- | --- |
| distance | Distance | 0.9456 | | | -0.0059 |  |
| **Gender** | Binary | -0.0392 | | | -0.1818 | Balanced, <0.2 |
| **Age** |  |  | | |  |  |
| <18y | Binary | -0.0556 | | | 0.0000 | Balanced, <0.2 |
| 18-35y | Binary | 0.1299 | | | -0.1818 | Balanced, <0.2 |
| 35-45 | Binary | -0.1046 | | | 0.0909 | Balanced, <0.2 |
| 45-60 | Binary | 0.0964 | | | 0.0909 | Balanced, <0.2 |
| ≥60y | Binary | -0.0662 | | | 0.0000 | Balanced, <0.2 |
| **BMI (kg/m^2^)** |  |  | | |  |  |
| <18.5 | Binary | -0.0286 | | | -0.0909 | Balanced, <0.2 |
| 18.5-25 | Binary | 0.0253 | | | 0.1818 | Balanced, <0.2 |
| ≥25 | Binary | 0.0033 | | | -0.0909 | Balanced, <0.2 |
| **Resistance category** |  |  | | |  |  |
| RR-TB | Binary | 0.0310 | | | -0.0909 | Balanced, <0.2 |
| MDR-TB | Binary | -0.0596 | | | 0.0909 | Balanced, <0.2 |
| pre-XDR-TB | Binary | 0.0286 | | | 0.0000 | Balanced, <0.2 |
| **Treatment times (mo)** | Contin. | 0.2598 | | | -0.0286 | Balanced, <0.2 |
| Cavities | Binary | 0.2304 | | | 0.0000 | Balanced, <0.2 |
| Cycloserine | Binary | 0.0735 | | | 0.0909 | Balanced, <0.2 |
| Amikacin/Capreomycin | Binary | 0.2304 | | | 0.0000 | Balanced, <0.2 |
| Prothionamide | Binary | -0.2721 | | 0.0000 | | Balanced, <0.2 |
| Balance tally for mean differences | Count |  | | | | |
| Balanced, <0.2 | 18 |  |  |  |  |  |
| Not Balanced, >0.2 | 0 |  |  |  |  |  |
| Sample sizes | Control | Treated |  | | | |
| All | 72 | 17 |  |  |  |  |
| Matched | 11 | 11 |  |  |  |  |
| Unmatched | 61 | 6 |  |  |  |  |

Diff.Un: differences of unadjusted, Diff.Adj: differences of adjusted, M.Threshold: threshold of match, mo: month. BMI: body mass index, RR-TB: rifampicin-resistant tuberculosis, MDR: multidrug-resistant tuberculosis, pre-XDR: pre-extensively drug-resistant tuberculosis.

**Table S13. Baseline comparison between the group that met the criteria for bedaquiline prolongation but did not receive it and prolonged group before and after PSM**

| Variable | Number (%) (Unadjusted) | | Unadjusted | | Number (%) (Adjusted) | | Adjusted | |
| --- | --- | --- | --- | --- | --- | --- | --- | --- |
|  | 6mo (N=35) | >12mo (N=88) | *P* | SMD | 6mo (N=22) | >12mo (N=22) | *P* | SMD |
| **Gender (Male)** | 24 ( 68.6) | 59 (67.0) | 0.871 | 0.033 | 7 (31.8) | 7 (31.8) | 1.000 | <0.001 |
| **Age** | | | | | | | | |
| <18y | 2 (5.7) | 3 (3.4) | 0.305 | 0.410 | 1 (4.5) | 2 (9.1) | 1.000 | <0.001 |
| 18-35y | 13 (37.1) | 47 (53.4) |  |  | 9 (40.9) | 10 (45.5) |  |  |
| 35-45y | 8 (22.9) | 15 (17.0) |  |  | 5 (22.7) | 4 (18.2) |  |  |
| 45-60y | 7 (20.0) | 18 (20.5) |  |  | 5 (22.7) | 4 (18.2) |  |  |
| ≥60y | 5 (14.3) | 5 (5.7) |  |  | 2 (9.1) | 2 (9.1) |  |  |
| **BMI** (kg/m^2^**)** | | | | | | | | |
| <18.5 | 10 (28.6) | 15 (17.0) | 0.281 | 0.340 | 7 (31.8) | 4 (18.2) | 0.488 | 0.319 |
| 18.5-25 | 24 (68.6) | 66 (75.0) |  |  | 4 (18.2) | 18 (81.8) |  |  |
| ≥25 | 1 (2.9) | 7 (8.0) |  |  | 0 (0.0) | 0 (0.0) |  |  |
| **Diabetes** | 8 (22.9) | 11 (12.5) | 0.152 | 0.274 | 5 (22.7) | 2 (9.1) | 0.412 | 0.379 |
| **Resistance category** | | | | | | | | |
| RR-TB | 0 (0.0) | 11 (12.5) | **0.002** | 0.787 | 0 (0.0) | 0 (0.0) | 0.664 | 0.267 |
| MDR-TB | 5 (14.3) | 29 (33.0) |  |  | 4 (18.2) | 2 (9.1) |  |  |
| pre-XDR-TB | 30 (85.7) | 48 (54.5) |  |  | 18 (81.8) | 20 (90.9) |  |  |
| **Bronchial tuberculosis** | 7 (20.0) | 12 (13.6) | 0.378 | 0.171 | 4 (18.2) | 3 (13.6) | 1.000 | 0.125 |
| **Tuberculous pleurisy** | 3 (8.6) | 11 (12.5) | 0.755 | 0.128 | 1 (4.5) | 0 (0.0) | 1.000 | 0.309 |
| **Extrapulmonary** | 2 (5.7) | 3 (3.4) | 0.622 | 0.111 | 1 (4.5) | 1 (4.5) | 1.000 | <0.001 |
| **Treatment category** | | | | | | | | |
| New | 14 (40.0) | 23 (26.1) | 0.130 | 0.298 | 7 (31.8) | 11 (50.0) | 0.358 | 0.376 |
| Retreatment | 21 (60.0) | 65 (73.9) |  |  | 15 (68.2) | 11 (50.0) |  |  |
| **Culture at baseline (Positive)*** | 34 (97.1) | 78 (88.6) | 0.177 | 0.336 | 22 (100.0) | 22 (100.0) | 1.000 | <0.001 |
| **Clinical symptoms** | |  |  |  |  |  |  |  |
| Cough | 26 (74.3) | 48 (54.5) | **0.044** | 0.421 | 16 (72.7) | 13 (59.1) | 0.525 | 0.291 |
| Expectoration | 23 (65.7) | 41 (46.6) | **0.055** | 0.393 | 15 (68.2) | 12 (54.5) | 0.536 | 0.283 |
| Fever | 9 (25.7) | 15 (17.0) | 0.274 | 0.213 | 5 (22.7) | 2 (9.1) | 0.412 | 0.379 |
| Hemoptysis | 10 (28.6) | 14 (15.9) | 0.110 | 0.308 | 7 (31.8) | 3 (13.6) | 0.281 | 0.444 |
| Dyspnea | 4 (11.4) | 11 (12.5) | 0.870 | 0.033 | 2 (9.1) | 5 (22.7) | 0.412 | 0.379 |
| **Cavities** | 23 (65.7) | 48 (54.5) | 0.258 | 0.230 | 14 (63.6) | 13 (59.1) | 1.000 | 0.039 |
| **Scope of lesions (lobes)** | | | | | | | | |
| 1-2 | 9 (25.7) | 39 (44.3) | 0.143 | 0.408 | 8 (36.4) | 7 (31.8) | 1.000 | 0.138 |
| 3-4 | 11 (31.4) | 18 (20.5) |  |  | 3 (13.6) | 4 (18.2) |  |  |
| 5-6 | 15 (42.9) | 31 (35.2) |  |  | 11 (50.0) | 11 (50.0) |  |  |
| **Treatment times (mo) , median(IQR)** | 23.0 (18.50, 25.00) | 18.0 (15.00, 20.25) | **<0.001** | 0.686 | 20.0 (18.00, 23.75) | 19.0 (18.00, 22.25) | 0.531 | 0.007 |
| **Drugs** |  |  |  |  |  |  |  |  |
| Linezolid | 34 ( 97.1) | 77 (87.5) | 0.104 | 0.368 | 21 (95.5) | 20 (90.9) | 1.000 | 0.181 |
| Fluoroquinolone | 4 (11.4) | 21 (23.9) | 0.122 | 0.331 | 3 (13.6) | 4 (18.2) | 1.000 | 0.125 |
| Clofazimine | 30 (85.7) | 58 (65.9) | **0.028** | 0.475 | 20 (90.9) | 17 (77.3) | 0.412 | 0.379 |
| Cycloserine | 23 (65.7) | 68 (77.3) | 0.187 | 0.258 | 15 (68.2) | 15 (68.2) | 1.000 | <0.001 |
| Pyrazinamide | 16 (45.7) | 46 (52.3) | 0.512 | 0.131 | 8 (36.4) | 12 (54.5) | 0.364 | 0.371 |
| Ethambutol | 2 (5.7) | 6 (6.8) | 1.00 | 0.046 | 1 (4.5) | 3 (13.6) | 0.607 | 0.320 |
| Delamanid | 0 (0.0) | 3 (3.4) | 0.557 | 0.266 | 0 (0.0) | 0 (0.0) | 1.000 | <0.001 |
| Amikacin/Capreomycin | 18 (51.4) | 30 ( 34.1) | 0.075 | 0.356 | 10 (45.5) | 8 (36.4) | 0.759 | 0.186 |
| Sodium p-aminosalicylate | 16 (45.7) | 26 ( 29.5) | 0.088 | 0.338 | 11 (50.0) | 8 (36.4) | 0.543 | 0.278 |
| Prothionamide | 22 (62.9) | 48 ( 54.5) | 0.401 | 0.169 | 15 (68.2) | 13 (59.1) | 0.754 | 0.190 |

Data are n (%) or median (IQR), SMD: standard mean difference, BMI: body mass index, RR-TB: rifampicin-resistant tuberculosis, MDR: multidrug-resistant tuberculosis, pre-XDR: pre-extensively drug-resistant tuberculosis, mo: months, CT: computed tomography. Percentages calculated based on the number of patients with a result at baseline (baseline refers to 4 weeks before or 2 weeks after treatment initiation).

**Table S14. Correlation analysis of factors affecting treatment outcomes (group that met the criteria for bedaquiline prolongation but did not receive it and prolonged group)**

| Dependent: Outcome | | Success  (n=90) | Failure  (n=33) | OR (univariable)  (95% CI，*P*） |
| --- | --- | --- | --- | --- |
| **Gender** | | | | |
| Male | | 60 (66.7) | 23 (69.7) |  |
| Female | | 30 (33.3) | 10 (30.3) | 0.87 (0.37-2.06, *P*=0.751) |
| **Age** |  |  |  |  |
| <18y | | 4 (4.4) | 1 (3.0) |  |
| 18-35y | | 45 (50) | 15 (45.5) | 1.33 (0.14-12.88, *P*=0.804) |
| 35-45y | | 15 (16.7) | 8 (24.2) | 2.13 (0.20-22.44, *P*=0.528) |
| 45-60y | | 19 (21.1) | 6 (18.2) | 1.26 (0.12-13.59, *P*=0.847) |
| ≥60y | | 7 (7.8) | 3 (9.1) | 1.71 (0.13-22.51, *P*=0.682) |
| **BMI (kg/m^2^)** | | | | |
| <18.5 | | 15 (16.7) | 10 (30.3) |  |
| 18.5-25 | | 68 (75.6) | 22 (66.7) | 0.49 (0.19-1.23, *P*=0.129) |
| ≥25 | | 7 (7.8) | 1 (3.0) | 0.21 (0.02-2.02, *P*=0.178) |
| **Treatment times（mo), Mean ± SD** | | 18.5 ± 5.3 | 21.8 ± 7.7 | **1.09 (1.02-1.17, *P*=0.012)** |
| **Resistance category** | |  |  |  |
| RR-TB | | 9 (10) | 2 (6.1) |  |
| MDR-TB | | 28 (31.1) | 6 (18.2) | 0.96 (0.16-5.65, *P*=0.968) |
| pre-XDR-TB | | 53 (58.9) | 25 (75.8) | 2.12 (0.43-10.56, *P*=0.358) |
| **Bronchial tuberculosis** | | 14 (15.6) | 5 (15.2) | 0.97 (0.32-2.94, *P*=0.956) |
| **Tuberculous pleurisy** | | 9 (10) | 5 (15.2) | 1.61 (0.50-5.20, *P*=0.428) |
| **Extrapulmonary** | | 4 (4.4) | 1 (3.0) | 0.67 (0.07-6.24, *P*=0.727) |
| **Treatment category** | | | | |
| New | | 27 (30.0) | 10 (30.3) |  |
| Retreatment | | 63 (70.0) | 23 (69.7) | 0.99 (0.41-2.35, *P*=0.974) |
| **Diabetes** | | 13 (14.4) | 6 (18.2) | 1.32 (0.46-3.81, *P*=0.612) |
| **Culture at baseline (Positive)** | | 82 (91.1) | 30 (90.9) | 0.98 (0.24-3.92, *P*=0.972) |
| **Clinical symptoms** | |  |  |  |
| Cough | | 54 (60.0) | 20 (60.6) | 1.03 (0.45-2.32, *P*=0.952) |
| Expectoration | | 47 (52.2) | 17 (51.5) | 0.97 (0.44-2.16, *P*=0.945) |
| Fever | | 19 (21.1) | 5 (15.2) | 0.67 (0.23-1.96, *P*=0.462) |
| Hemoptysis | | 17 (18.9) | 7 (21.2) | 1.16 (0.43-3.10, *P*=0.773) |
| Dyspnea | | 12 (13.3) | 3 (9.1) | 0.65 (0.17-2.47, *P*=0.527) |
| **Cavities** | | 47 (52.2) | 24 (72.7) | **2.44 (1.02-5.83, *P*=0.045)** |
| **Scope of lesions (lobes)** | | | | |
| 1-2 | | 39 (43.3) | 9 (27.3) |  |
| 3-4 | | 20 (22.2) | 9 (27.3) | 1.95 (0.67-5.68, *P*=0.221) |
| 5-6 | | 31 (34.4) | 15 (45.5) | 2.10 (0.81-5.43, *P*=0.127) |
| **Drugs** | |  |  |  |
| Linezolid | | 80 (88.9) | 31 (93.9) | 1.94 (0.40-9.35, *P*=0.410) |
| Fluoroquinolone | | 19 (21.1) | 6 (18.2) | 0.83 (0.30-2.30, *P*=0.721) |
| Clofazimine | | 62 (68.9) | 26 (78.8) | 1.68 (0.65-4.32, *P*=0.284) |
| Cycloserine | | 69 (76.7) | 22 (66.7) | 0.61 (0.25-1.46, *P*=0.265) |
| Pyrazinamide | | 47 (52.2) | 15 (45.5) | 0.76 (0.34-1.70, *P*=0.506) |
| Ethambutol | | 4 (4.4) | 4 (12.1) | 2.97 (0.70-12.62, *P*=0.141) |
| Delamanid | | 3 (3.3) | 0 (0.0) | 0.00 (0.00-Inf, *P*=0.991) |
| Amikacin/Capreomycin | | 30 (33.3) | 18 (54.5) | **2.40 (1.06-5.41, *P*=0.035)** |
| Sodium p-aminosalicylate | | 31 (34.4) | 11 (33.3) | 0.95 (0.41-2.21, *P*=0.908) |
| Prothionamide | | 52 (57.8) | 18 (54.5) | 0.88 (0.39-1.96, *P*=0.749) |
| Data are n (%) or Mean **±** SD, SD: standard Deviation, OR: odds ratio, mo: month. BMI: body mass index, RR-TB: rifampicin-resistant tuberculosis, MDR: multidrug-resistant tuberculosis, pre-XDR: pre-extensively drug-resistant tuberculosis. | | | | |

**Table S15. Balance test of PSM (group that met the criteria for bedaquiline prolongation but did not receive it and prolonged group)**

|  | Type | Diff.Un | | Diff.Adj | M.Threshold |
| --- | --- | --- | --- | --- | --- |
| distance | Distance | 1.7047 | | 0.0582 |  |
| **Gender** | Binary | 0.0153 | | 0.0000 | Balanced, <0.2 |
| **Age** |  |  | |  |  |
| <18y | Binary | -0.0231 | | 0.0455 | Balanced, <0.2 |
| 18-35y | Binary | 0.1627 | | 0.0455 | Balanced, <0.2 |
| 35-45 | Binary | -0.0581 | | -0.0455 | Balanced, <0.2 |
| 45-60 | Binary | 0.0045 | | -0.0455 | Balanced, <0.2 |
| ≥60y | Binary | -0.0860 | | 0.0000 | Balanced, <0.2 |
| **BMI** |  |  | |  |  |
| <18.5 kg/m^2^ | Binary | -0.1153 | | -0.1364 | Balanced, <0.2 |
| 18.5-25 kg/m^2^ | Binary | 0.0643 | | 0.1364 | Balanced, <0.2 |
| ≥25 kg/m^2^ | Binary | 0.0510 | | 0.0000 | Balanced, <0.2 |
| **Resistance category** |  |  | |  |  |
| RR-TB | Binary | 0.1250 | | 0.0000 | Balanced, <0.2 |
| MDR-TB | Binary | 0.1867 | | -0.0909 | Balanced, <0.2 |
| pre-XDR-TB | Binary | -0.3117 | | 0.0909 | Balanced, <0.2 |
| **Treatment category** | Binary | 0.1386 | | 0.0077 | Balanced, <0.2 |
| Cough | Binary | -0.1974 | | -0.1364 | Balanced, <0.2 |
| Expectoration | Binary | -0.1912 | | -0.1364 | Balanced, <0.2 |
| **Treatment times (mo)** | Contin. | -0.6854 | | 0.0077 | Balanced, <0.2 |
| **Cavities** | Binary | -0.1117 | | -0.0455 | Balanced, <0.2 |
| Scope of lesions (1-2) | Binary | 0.1860 | | -0.0455 | Balanced, <0.2 |
| Scope of lesions (3-4) | Binary | -0.1097 | | 0.0455 | Balanced, <0.2 |
| Scope of lesions (5-6) | Binary | -0.0763 | | 0.0000 | Balanced, <0.2 |
| Cfz | Binary | -0.1981 | | -0.1364 | Balanced, <0.2 |
| Cm/Am | Binary | -0.1734 | | -0.0909 | Balanced, <0.2 |
| Balance tally for mean differences | Count |  | | | |
| Balanced, <0.2 | 23 |  |  |  |  |
| Not Balanced, >0.2 | 0 |  |  |  |  |
| Sample sizes | Control | Treated |  | | |
| All | 35 | 88 |  |  |  |
| Matched | 22 | 22 |  |  |  |
| Unmatched | 13 | 66 |  |  |  |

Diff.Un: differences of unadjusted, Diff.Adj: differences of adjusted, M.Threshold: threshold of match, mo: month. BMI: body mass index, RR-TB: rifampicin-resistant tuberculosis, MDR: multidrug-resistant tuberculosis, pre-XDR: pre-extensively drug-resistant tuberculosis.
